# A multiscale accuracy assessment of moisture content predictions using time-lapse electrical resistivity tomography in mine tailings

**DOI:** 10.1038/s41598-023-48100-w

**Published:** 2023-11-27

**Authors:** Adrien Dimech, Anne Isabelle, Karine Sylvain, Chong Liu, LiZhen Cheng, Bruno Bussière, Michel Chouteau, Gabriel Fabien-Ouellet, Charles Bérubé, Paul Wilkinson, Philip Meldrum, Jonathan Chambers

**Affiliations:** 1 Université du Québec en Abitibi-Témiscamingue (UQAT), Rouyn Noranda, QC J9X 5E4 Canada; 2 Polytechnique Montréal, Montréal, QC H3T 1J4 Canada; 3 Research Institute of Mines and Environment (RIME), Montréal, QC Canada; 4 British Geological Survey (BGS), Environmental Science Centre, Keyworth, Nottingham, NG12 5GG UK

**Keywords:** Environmental impact, Geophysics, Hydrogeology, Environmental sciences, Engineering

## Abstract

Accurate and large-scale assessment of volumetric water content (VWC) plays a critical role in mining waste monitoring to mitigate potential geotechnical and environmental risks. In recent years, time-lapse electrical resistivity tomography (TL-ERT) has emerged as a promising monitoring approach that can be used in combination with traditional invasive and point-measurements techniques to estimate VWC in mine tailings. Moreover, the bulk electrical conductivity (EC) imaged using TL-ERT can be converted into VWC in the field using petrophysical relationships calibrated in the laboratory. This study is the first to assess the scale effect on the accuracy of ERT-predicted VWC in tailings. Simultaneous and co-located monitoring of bulk EC and VWC are carried out in tailings at five different scales, in the laboratory and in the field. The hydrogeophysical datasets are used to calibrate a petrophysical model used to predict VWC from TL-ERT data. Overall, the accuracy of ERT-predicted VWC is $\pm 0.03~\textrm{m}^3/\textrm{m}^3$, and the petrophysical models determined at sample-scale in the laboratory remain valid at larger scales. Notably, the impact of temperature and pore water EC evolution plays a major role in VWC predictions at the field scale (tenfold reduction of accuracy) and, therefore, must be properly taken into account during the TL-ERT data processing using complementary hydrogeological sensors. Based on these results, we suggest that future studies using TL-ERT to predict VWC in mine tailings could use sample-scale laboratory apparatus similar to the electrical resistivity Tempe cell presented here to calibrate petrophysical models and carefully upscale them to field applications.

## Introduction

Accurate estimation of the spatial distribution of volumetric water content (VWC) is important for understanding most hydrogeological processes in the vadose zone^1,2^. Among others fields of application, the spatio-temporal dynamics of VWC are key parameters for (i) weather and climate predictions^3–5^, (ii) moisture-induced landslides, floods and droughts prevention^6–11^, (iii) agricultural and irrigation scheduling^2,12–15^, (iv) infrastructure stability assessment^16,17^ and (v) water resource management^18–20^. As discussed by Bussière et al.^21^ and Dimech et al.^22^, VWC monitoring is also a key component for monitoring programs of tailings storage facilities (TSF) and waste rock piles (WRP), which typically extend across several $\textrm{km}^2$ and can be highly heterogeneous^23,24^. Indeed, most of the geotechnical and geochemical stability issues in WRP and TSF are closely connected to (i) moisture content distribution (e.g., Power, Ramasamy, and Mkandawire^25–27^) and/or (ii) water infiltration, seepage and evapo-transpiration (e.g.,^28–31^). In addition, several mining waste reclamation approaches such as covers with capillary barrier effects (CCBE) rely on VWC in moisture-retaining layers to control the oxygen migration from the atmosphere, which limits the risk of acid mine drainage (AMD) generation^32,33^.

Classical methods for measuring VWC in the field are divided into two categories with different spatial extent (i.e., overall coverage of measurements) and spatial support (i.e., integration volume or area of measurements)^3,34,35^. On the one hand, point sensing instrument networks such as time-domain reflectometry or capacitance sensors are known to measure VWC accurately but locally (typically a few centimeters around the sensors) with limited perturbation of the subsurface, as opposed to destructive sampling^2^. On the other hand, remote sensing approaches (passive and active, using aerial measurements or satellite) allow estimating VWC across large-scale areas without subsurface perturbation. Reported spatio-temporal resolutions range from 10 to $500~\text {m}$^35,36^ with a revisit time as low as a few days^37^. However, these methods have a shallow penetration depth (generally less than ten centimeters)^3,35^. Geophysical approaches have been identified as promising monitoring tools to fill the gap between local and surface VWC observations since they aim to recover the distribution of physical properties in the subsurface, from centimetric to kilometric scales^18,38–43^.

Time-lapse electrical resistivity tomography (TL-ERT) is one of the most effective geophysical methods for the characterization of VWC dynamics^44–46^. TL-ERT can be used to image the distribution of subsurface bulk electrical conductivity (referred to as EC), whose spatio-temporal changes have been extensively used as a proxy for VWC changes in various contexts over the past 30 years (e.g., hydrogeothermal, environmental, geotechnical and ecological monitoring, following the classification of TL-ERT studies proposed by Dimech et al.^22^). Recently, particular attention has been given to the quantitative interpretation of TL-ERT results, for instance through the conversion of bulk EC into VWC, temperature or contaminant concentration (e.g.,^47–49^. Nonetheless, assessing VWC from TL-ERT can be challenging due to the many physical parameters also affecting subsurface bulk EC (such as temperature, pore water EC, porosity, grain size distribution and mineralogy^50,51^). In this context, some studies have assessed the accuracy of the ERT-predicted VWC, for example, by comparing them with conventional gravimetric methods applied on samples collected on the field^52–54^ or hydrogeological data from sensors installed on the field^2,55–58^.

Most studies aiming to predict VWC using TL-ERT at the field scale rely on petrophysical relationships connecting bulk EC and VWC, which are generally determined from small-scale disturbed samples in the laboratory (e.g.,^59,60^). However, several studies mention that a sample-size laboratory characterization may not be representative of field conditions^61^. For example, (i) the presence of macropores, desiccation cracks or bedding planes in the sample, (ii) small-scale heterogeneity or (iii) multiphasic composition of the material could have greater influence at sample scale, as opposed to field scale measurements, which capture the bulk electrical properties averaged across larger volumes^61–65^.

As detailed by Dimech^66^, mining wastes can be challenging media for the application of TL-ERT since they are generally remote, complex sites and in constant evolution with harsh field conditions, such as landfill sites for instance^67^. In addition, TL-ERT may be applied to characterize VWC dynamics with high spatio-temporal resolution for long time periods and across large scales and many physical parameters affecting bulk EC may evolve simultaneously in the mining wastes, such as VWC, temperature and pore fluid EC for instance^66^. Nonetheless, few studies have focused on the accuracy of VWC estimation in mine tailings using TL-ERT. Moreover, few studies have investigated the validity of laboratory-determined petrophysical relationships up-scaling from sample-scale to laboratory and/or field scale applications, especially in mining wastes. However, these two aspects are crucial to successful applications of TL-ERT for VWC monitoring in mining wastes. Indeed, high accuracy of predicted VWC and better understanding of uncertainties are usually needed for geotechnical and geochemical stability monitoring of mining wastes at field scale^21,22,68,69^. This study aims to (i) develop a methodology for calibrating petrophysical relationships between bulk EC and VWC in mine tailings, both at sample-scale and at larger scales and (ii) assess the accuracy of the VWC predicted by ERT using the petrophysical relationships specific to each experiment, and using the relationships determined at different scales to study the scaling impact on petrophysical models. Quantifying the accuracy of ERT-predicted VWC in mine tailings will aid in supporting future monitoring studies using TL-ERT as a complementary tool to measure VWC, along with conventional hydrogeological methods. Moreover, developing strategies to up-scale petrophysical relationships accurately will allow the recovery of complex spatio-temporal dynamics of VWC in mine tailings across large scales, which may not be possible when solely relying on point data.

## Materials and methods

### Site description and materials

The study site is located at Canadian Malartic mine, a world-class, large tonnage and low-grade intrusion-related gold deposit located in Québec, Canada^70^. Gold mineralization is hosted mainly in meta sedimentary rocks (metaturbidite) and porphyritic intrusions (quartz-monzodiorite)^71^, with a mean grade of 1.07 $\mathrm {g/t}$ Au^72^ and an estimated total gold resource exceeding 10 million ounces^73^. Four large-scale experimental multi-layer covers were built in 2019 and 2020 to identify the future reclamation of the TSF covering nearly $6~\text {km}^2$. The 300 m-long, 10 m-large and 2.3 m-high covers are expected to provide valuable information at the pilot scale and under real meteorological conditions to guide the design of TSF reclamation^74^. This study focuses on the experimental CCBE made of waste rocks, tailings and overburden, whose role is to reduce the downward oxygen flux from the atmosphere to the tailings, thanks to the high VWC of a 1 m-thick moisture-retaining layer made of compacted tailings^33,75^.

**Figure 1:**
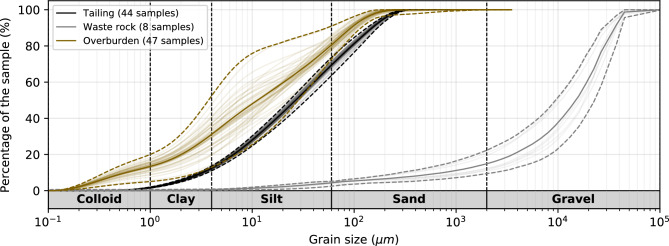
Grain size distribution of the materials used in the experiment. In total, 44 different samples of tailings (black), 8 samples of waste rocks (grey) and 47 samples of overburden (brown) have been analyzed. Each individual grain size distribution is shown using shaded lines. The mean, minimum and maximum grain size distributions are shown in solid line and dashed lines.

The materials used for this study were sampled from 2017 to 2020 at Canadian Malartic mine. As shown in Fig. 1, the tailings consist of finely milled rocks with nearly 80 % of particles ranging from 2$\upmu \hbox {m}$ to 80$\upmu \hbox {m}$ (i.e., mostly silt), $1.22~\pm ~0.08~\%$ sulfide content, $0.55~\pm ~0.03~\%$ carbon content ($n=44$ samples), and some trace metals (see^76–78^ for more details). The 44 tailings samples collected for grain-size, sulfide and carbon content analysis have similar properties, which demonstrates the overall homogeneity of tailings. The grain size distribution of overburden (47 samples) and waste rocks (8 samples) are also presented in Fig. 1 in brown and grey, respectively. The overburden material is finer than the tailings (mostly clay and silt) and a greater variability is observed between each sample (e.g., $D_{60}=20.9~\pm ~7.8 \upmu \hbox {m}$ for overburden and $D_{60}=40.6~\pm ~3.2~\upmu \hbox {m}$ for tailings). Finally, the waste rocks used in this study mostly consist of gravel and sand, with nearly 5 % of particles smaller than 100$\upmu \hbox {m}$.

### Experimental setups

**Figure 2:**
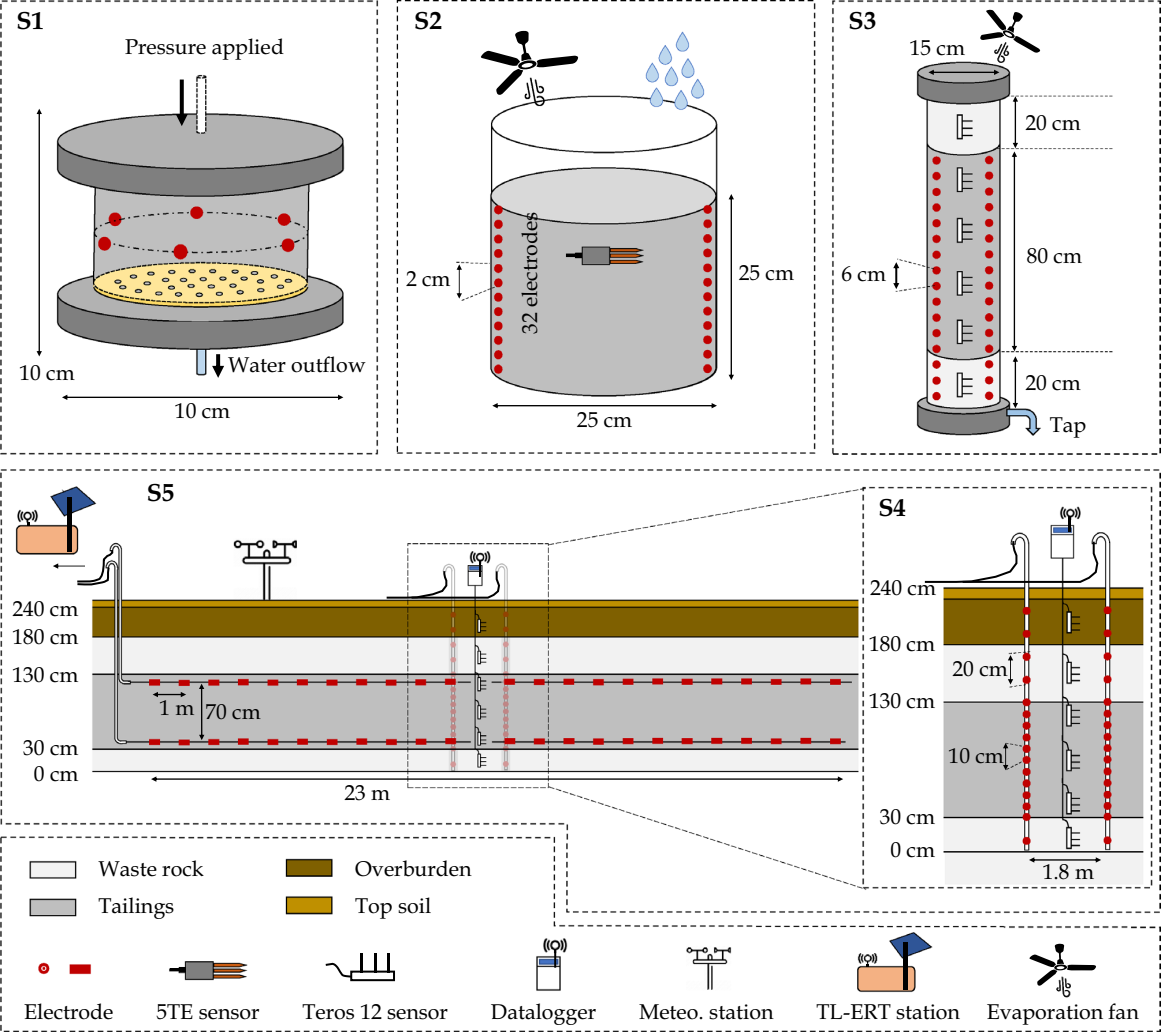
Illustration of the five scales investigated in this study to assess petrophysical relationships in mine tailings in the laboratory (S1—cell scale, S2—bucket scale and S3—column scale), and in the field (**S4** - local scale and **S5** - pilot scale).

The relationship between VWC measured by hydrogeological sensors and the bulk EC imaged using TL-ERT measurements is investigated at five different scales, summarized in Fig. 2. The size of these setups ranges from a few centimeters in the laboratory (**S1**, **S2** and **S3**) to several meters in the field (**S4** and **S5**). The same procedure was followed for the preparation and installation of the tailings for each experimental setup to ensure maximum comparability between the different scales. In the laboratory, the tailings material is dried using an oven at $60~^{\circ }\textrm{C}$. The dry material is then deagglomerated into a fine powder (without changing the grain size distribution) and homogenized. A predetermined volume of deionized water is then added to the dry samples to reach an initial VWC of 0.40 $\textrm{m}^3/\textrm{m}^3$, which corresponds to the full saturation of the sample, knowing the dry density of the tailings ($2.68\pm 0.01~\textrm{g}/\textrm{cm}^3$, $n=44$ samples) and a target porosity of $\phi =0.4$. Successive 5 cm-high layers of wet tailings samples are compacted inside the laboratory cells at the needed porosity to ensure homogeneity of the material compaction. In the field, the wet tailings were compacted using a power shovel and the porosity of successive 25 cm-high layers was measured in-situ using a nucleodensimeter ($\phi =0.40\pm 0.02$, $n=73$ measurements), which is consistent with previous experimental cells built at Canadian Malartic mine (e.g.,^78^).

#### Scale 1: electrical resistivity tempe cell

**Figure 3:**
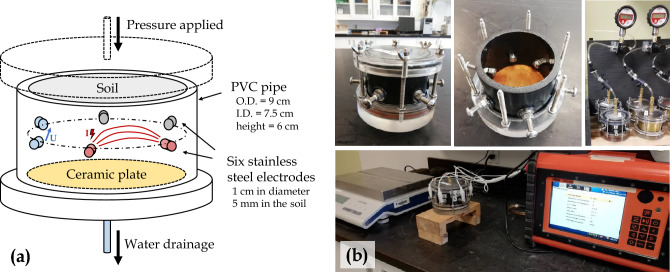
(**a**) Schematic view of the electrical-resistivity tempe cell (ER-TC) used in the experiment. (**b**) Photographs showing the ER-TC, the porous ceramic plate, the six stainless steel electrodes and the ABEM Terrameter LS resistivity meter.

Figure 3 presents a new laboratory apparatus inspired from Tempe cells^79^ designed to simultaneously recover VWC and bulk EC within a soil sample under different pressure conditions, hence referred to as electrical resistivity Tempe cell (ER-TC). A Tempe cell (model 1405 - SoilMoisture Equipment) is modified by including six stainless steel electrodes to carry out ERT measurements during drying of the tailings sample. The dimension of the electrodes (width and length indicated on Fig. 3) and their location in the cylindrical cell are determined following the practical guidelines discussed by Lee and 98 Santamarina^80^ and Clement and Moreau^81^. The conventional brass cylinder is replaced by a PVC pipe that can withstand pressures of up to $350~\textrm{kPa}$. 15 different steps of pressure are then applied to the top of the cell using air pressure controllers and nitrogen high-pressure bottles to drive changes in VWC^79,82^. The positive pressure applied on the top of the cell allows mimicking a corresponding suction which would be applied at the bottom of the cell in the vadose zone^83^. The ER-TCs are weighed after two days at each pressure increment to calculate VWC in the sample. Pore water EC is also monitored by sampling the water coming out of the cell. In the meantime, ERT monitoring is carried out to recover bulk EC variations in the ER-TC using a Terrameter LS (ABEM).

#### Scale 2: laboratory bucket

**Figure 4:**
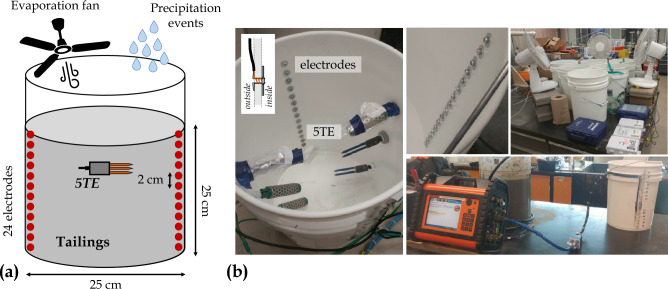
(**a**) Schematic view of the laboratory bucket used in the experiment. (**b**) Photographs show the electrodes and 5TE sensor location in the bucket as well as the monitoring devices (EM50 dataloggers and ABEM Terrameter LS). Several fans were used to accelerate evaporation and precipitation events were carried out to mimic realistic meteorological conditions.

Figure 4 presents the laboratory bucket used to monitor the temporal variations of VWC and bulk EC in tailings under controlled cycles of wetting and drainage^84^. A 30 cm-high, 25 cm-diameter bucket is filled with compacted tailings and instrumented with a 5TE sensor (measuring VWC, bulk EC and temperature every 15 minutes) and two vertical profiles of 12 stainless-steel electrodes each. Each electrode is made of a 1 cm-diameter flat washer fixed by a 5 mm-diameter rivet, with a vertical spacing of 2 cm. As presented by Sylvain, Pabst, and Dimech^84^, several simulated precipitation events were carried out using deionized water to mimic precipitations ($50~\textrm{mm}$ per simulated precipitation event) and three fans were installed to accelerate evaporation in the tailings. Each wetting and drainage cycle lasted approximately one week and the hydrogeophysical monitoring was performed during two weeks in November 2018. TL-ERT monitoring was carried out using a Terrameter LS and approximately 150 ERT datasets were recorded, with a greater temporal resolution during simulated precipitation events (highest measurement rate of 12 ERT data acquisitions per hour).

#### Scale 3: laboratory column

**Figure 5:**
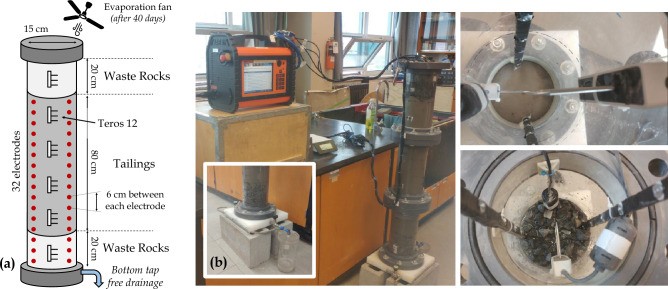
(**a**) Schematic view of the laboratory column used in the experiment. (**b**) Photographs showing the different steps of column assembling, the connection of the Teros 12 hydrogeological sensors to the ZL6 datalogger and the connection of electrodes to Terrameter LS.

Figure 5 presents the laboratory column used to reproduce the field-scale experimental CCBE under controlled conditions in the laboratory, as discussed by Isabelle^85^. A 120 cm-high, 15 cm-diameter Plexiglas column is assembled and instrumented to simultaneously monitor VWC and bulk EC. Two layers of waste rocks (20 cm-high each) are installed at the bottom and the top of the column and a 80 cm-high compacted tailings layer, which mimic the geometry of the CCBE built on the field. Initially, the column was fully saturated with deionized water. After a few days, the bottom tap was opened and the materials drained during five months, with an additional evaporation fan installed after 40 days to accelerate the decrease of moisture content in the column^85^. Six Teros 12 hydrogeological sensors are installed along the flanks of the columns to measure VWC, bulk EC and temperature every 5 min. In addition, two vertical profiles of 16 stainless-steel circular electrodes are installed in the column and connected to a Terrameter LS to carry out ERT monitoring during the free drainage (50 datasets). Each electrode is made of a 1 cm-diameter flat washer fixed by a 5 mm-diameter rivet and the vertical electrode spacing is 6 cm.

#### Scales 4 and 5: field experimental cover

**Figure 6:**
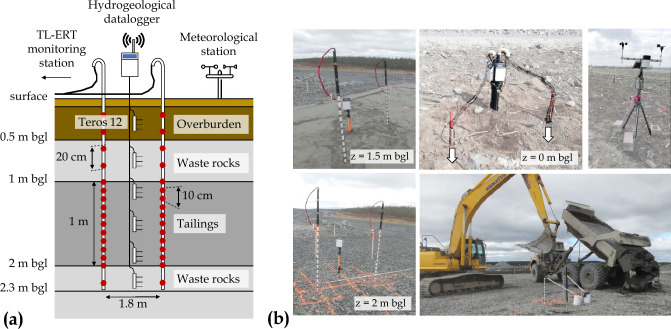
(**a**) Schematic description of the experimental cover at local scale. Location of hydrogeophysical monitoring instruments (moisture sensors and electrodes in red). (**b**) Photographs show the cover construction and instrument installation within the different materials. The meteorological station and the hydrogeological dataloggers at the surface are also shown (top right).

**Figure 7:**
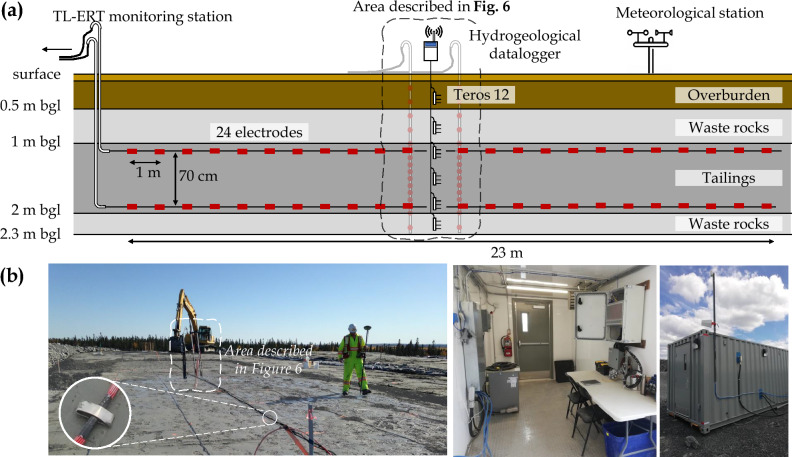
(**a**) Schematic description of the pilot-scale experimental cover and location of hydrogeophysical monitoring instruments. The electrodes along horizontal profiles in the tailings are represented in red. (**b**) Photographs show the cover construction, electrode design and instrument installation within the different materials. The bottom right photographs show the PRIME instrument located in a container which is used to carry out autonomous remote hydrogeophysical monitoring.

Figures 6 and 7 present the internal composition of the $2.3~\text {m}$-high experimental CCBE constructed at Canadian Malartic mine. The $1~\text {m}$-thick layer of compacted tailings is expected to behave as a moisture-retaining layer since capillary barrier effects are likely to develop at the boundary between the tailings and the waste rock layers, at the bottom (30 cm-thick) and at the top (50 cm-thick). The capillary barrier effects would then reduce both the downward water percolation and the upward water evaporation from the tailings, hence maintaining a high degree of saturation within the tailings layer. Finally, a 50 cm-thick layer made of overburden and topsoil is placed on the top of the waste rocks to promote vegetation development. Six Teros 12 sensors are installed along a vertical profile at the center of the CCBE and a ZL6 datalogger allows to remotely transfer VWC, bulk EC and temperature measurements for each layer (regular measurement rate of 30 min). At local scale (**S4**), two vertical profiles of 16 electrodes each, separated by 1.8 m, are installed around the profile of Teros 12. Each electrode is made of a 4 cm-diameter and 2.5 cm-long stainless steel cylinder fixed on a PVC pipe using a stainless-steel rivet. The centers of the electrodes are separated by a distance of 10 cm in the tailings and 20 cm elsewhere. In addition, at the pilot scale (**S5**), two 23 m-long horizontal profiles are installed inside the tailings layer, 15 cm away from the interfaces with waste rocks. Each parallel profile contains 24 rectangular stainless steel electrodes measuring 6 cm x 2.5 cm and separated by 1 m, which allows monitoring bulk EC in the tailings layer along the 23 m-long profiles. All the electrodes are connected to a PRIME system instrument^86,87^, which carries out ERT images four times a day (since the beginning of May 2021, still ongoing in 2023). The PRIME system itself is installed in a cabin powered by an electric line and equipped with an antenna and a router to allow autonomous transfer of TL-ERT data to remote servers. A meteorological station is installed at the surface of the cover to monitor air temperature and precipitations as shown in the top right panel of Fig. 6.

### Measurements and data processing

#### Meteorological and hydrogeological data

**Figure 8:**
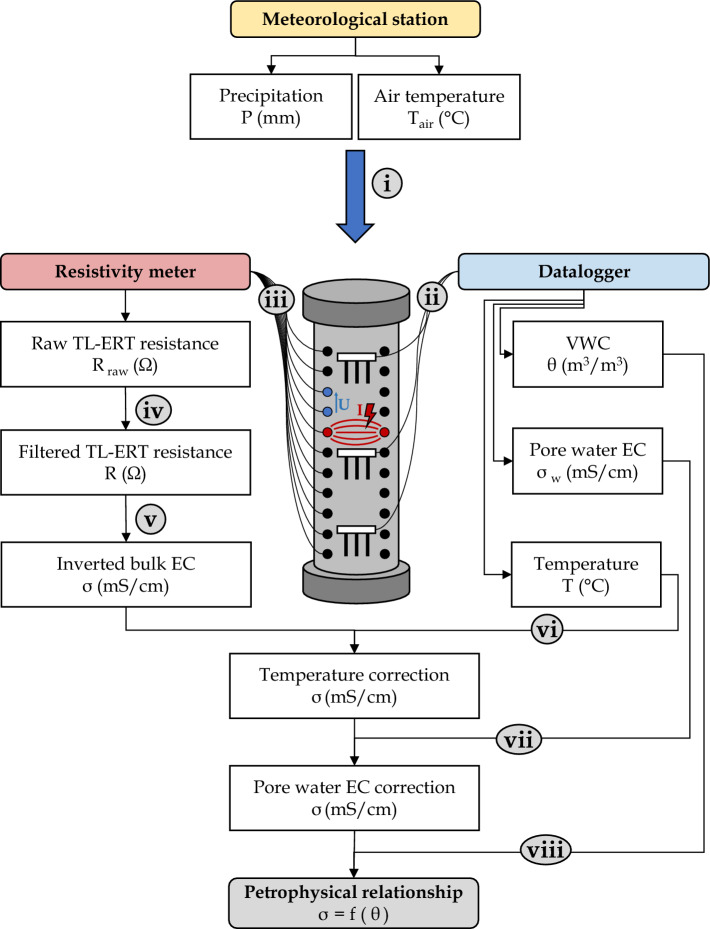
Workflow of hydrogeophysical data acquisition and processing used to recover petrophysical relationships from multi-scale experimental setups. The laboratory column (**scale 3**) is used as an example.

Figure 8 summarizes graphically the main steps proposed in this study to determine petrophysical relationships from the simultaneous and co-located monitoring of VWC and bulk EC at different scales. For all experiments, air temperature and precipitation are monitored using a meteorological station in the field or temperature probes for laboratory experiments (identified as step **(i)** in Fig. 8). 5TE or Teros 12 hydrogeological sensors are connected to dataloggers to monitor VWC, bulk EC and temperature (step **(ii)** in Fig. 8), which allows estimating interstitial pore water EC (see^30,88^). These capacitance sensors measure the dielectric permittivity of the medium using a high-frequency oscillating electrical voltage (i.e., $70~\textrm{MHz}$), which is then converted into VWC using Topp’s equation and material-specific calibrations^89,90^. For these sensors, typical resolution and accuracy of VWC measurements are respectively 0.001 $\textrm{m}^3/\textrm{m}^3$ and ± 0.02 $\textrm{m}^3/\textrm{m}^3$ when the proper calibrations are applied. As mentioned by Hen-Jones et al.^53^, the hydrogeological sensors use a two-points measurement to monitor bulk EC, which makes them more sensitive to changes in contact resistance than the four-points measurements done by ERT (especially at low moisture content).

#### Geophysical data acquisition

The electrodes installed along vertical or horizontal profiles are connected to two different resistivity meters (step **(iii)** in Fig. 8). The **Terrameter LS** commercialized by ABEM is used for laboratory-scale experiments. The **PRIME** instrument (*PRoactive Infrastructure Monitoring and Evaluation system*) designed by the British Geological Survey^86,87^ is used for continuous monitoring of the field-scale experiment. Both resistivity meters allow to carry out several potential measurements simultaneously according to specific four-electrodes measurement sequences, referred to as protocols (see Figure A.1 in Supplementary Material for an example of some protocols used). All quadrupoles are used for the ER-TC (scale **S1**), which corresponds to 45 direct measurements and 45 reciprocal measurements (i.e., configurations where current and potential electrodes are exchanged). For all other experimental setups, which contained more electrodes (scales **S2** to **S5**), dipole-dipole and Wenner-alpha configurations are used with *a* and *n* values between 1 and 3 (see^91–93^ for details). Since all experimental setups have two parallel lines of electrodes, the same ERT protocols (presented on Fig. A.1) are carried out at each scale. Reciprocal measurements are integrated into all TL-ERT data acquisition protocols following the guidelines of^94^, since the combination of direct and reciprocal measurements allows data error to be better estimated. Finally, special attention is given to the electrode polarisation issues by ensuring that the same electrode is not used for potential measurements just after being used to inject current^95^. The temporal resolution of TL-ERT data acquisition is adapted for each experimental setup. For example, more ERT datasets were recorded for laboratory experiments when the fastest changes of VWC were expected. For the field experiments, ERT datasets are collected with a regular sampling of four data collection per day.

#### Geophysical data processing

For each experimental setup, the apparent resistivity time-series are analyzed in order to identify and remove erroneous measurements, outliers, or unstable noisy data following the methodology described by Tso et al.^94^ and others^16,96^ (step **(iv)** in Fig. 8). Reciprocal errors are estimated for each four-electrodes measurement by calculating the difference between direct and reciprocal configurations and data with a reciprocal error greater than 10 % are removed^97^. Moreover, filtering is applied on the stacking error ($\epsilon ~<~10~\%$), the injected current intensity ($0.001~\textrm{mA}~<~I~<600~\textrm{mA}$) and the contact resistance between current electrodes ($R~<~10~000~\Omega$). A similar data processing approach is applied to the field data collected with the PRIME instrument, which yields to 400 000 valid measurements in total from May to November 2021. Nearly 85 % of the resulting filtered field dataset exhibits a reciprocal error lower than 1 %, which denotes satisfying data quality for the geoelectrical monitoring of tailings. Following the methodology proposed by^98,99^ and^94^, an envelope fit error model of the form $\epsilon_{{\rm R}} = a \cdot R + b$ is determined for each experiment, where $\epsilon_{{\rm R}}$ is the difference between direct and reciprocal resistance measurements, *a* ranges from 0.01 to 0.05 depending on the ERT data quality of each experiment and *b* equals to $0.001~\Omega$.

#### Geophysical data inversion

For each experimental setup, the filtered resistance data ($\textbf{d}$) is inverted using **pyGIMLi/BERT**^100–102^ to recover the distribution of bulk EC ($\textbf{m}$) (step **(v)** in Fig. 8). 3D models are used for laboratory setups given the cylindrical shape of the ER-TC, column or bucket whereas 2D vertical planes are defined for field experiments, given the lateral extension of the experimental cover. The inverted bulk EC distribution ${\textbf {m}}$ is assumed to be the best trade-off between two cost functions; the data misfit constraint and the model regularization constraint. The resulting cost-function (denoted as $\Phi ({\textbf {m}})$) is then expressed by^103^:

1 $$\begin{aligned} \Phi ({\textbf {m}}) = \left\| {\textbf {W}}_{{\rm d}} \cdot (d - \textit{F}({\textbf {m}}) )\right\| + \lambda \left\| {\textbf {W}}_{{\rm m}} \cdot ({\textbf {m}} - {\textbf {m}}_{0}) \right\| \end{aligned}$$

In this equation, $\textit{F}({\textbf {m}})$ corresponds to the forward modeling operator, which outputs a set of synthetic resistance data for a given distribution of bulk EC^101^. ${\textbf {W}}_{{\rm d}}$ and ${\textbf {W}}_{{\rm m}}$ are respectively the data weighting and the model constraint matrices and $\lambda$ is a regularization coefficient^103^. Finally, ${\textbf {m}}_{0}$ is a prior model that can be used to constraint the inversion.

For all experimental setups, an anisotropic spatial smoothing is applied to the distribution of bulk EC to reproduce the horizontal layering in VWC observed for unsaturated conditions (sharper changes of VWC in *z* than in *x* and *y*). Moreover, the geometry of the experimental cover is taken into account for the inversion of field data and horizontal layers are included in the 2D model. In addition, a specific spatial constraint was applied to reduce the smoothing between two neighboring layers, hence allowing sharp variations at the boundaries between different materials (e.g.,^30,103,104^). Moreover, the mesh was refined around sensors and electrodes to ensure that the cell size was much smaller than the volume of investigation of the hydrogeological sensors (approximately 5 cm around each sensor). The resulting meshes contain between 4 000 and 17 000 tetrahedron elements for 3D meshes and between 4 000 and 12 000 triangular cells for 2D meshes, which allows to perform each inversion in less than one minute. A temporal regularization is also applied for all experiments by using the previous inverted distribution of bulk EC as a starting model for each time step. This approach, referred to as the “cascade inversion”, helps limiting the unrealistic and erratic evolution of inverted bulk EC, which could be observed in some areas poorly constrained by the inversion process^47,105^.

#### Temperature and pore water EC corrections

After the inversion process, the inverted bulk EC values are extracted from the 3D or 2D models at the location of the hydrogeological sensors, knowing their volume of investigation. The average bulk EC of the corresponding cells is calculated for each sensor location. As illustrated by step **(vi)** in Fig. 8, a temperature correction is applied to the extracted inverted bulk EC. This allows to compare the laboratory experiments ($\simeq 23~^{\circ }\textrm{C}$) and the field experiment (between $0~^{\circ }\textrm{C}$ in Winter and $25~^{\circ }\textrm{C}$ in Summer), and to account for temperature variations. For all experiments, a standard temperature of $T_{\textrm{std}} = 25~^{\circ }\textrm{C}$ is used, and the temperature-corrected bulk EC $\sigma _{\textrm{corr}}$ is calculated by^106^:

2 $$\begin{aligned} \sigma _{\textrm{corr}} = \sigma \cdot \left[ \frac{1}{1 + tc \cdot (T-T_{\textrm{std}})} \right] \end{aligned}$$

where $\sigma$ is the bulk EC at the temperature *T*, measured by the hydrogeological sensors and *tc* is the temperature correction factor, which corresponds to the fractional change in $\sigma$ per degree Celsius. A value of $tc = 0.02~^{\circ }\textrm{C}^{-1}$ is used in this study, which means that bulk EC increases by 2% for a temperature increase of $1~^{\circ }\textrm{C}$^107–109^. It should be noted that the temperature measured by the hydrogeological sensors at different depths are assumed to be laterally constant; the vertical profiles of measured temperature are horizontally extrapolated to the imaging domain for the temperature correction of inverted bulk EC.

Similarly, a pore water EC correction is applied (step **(vii)** on Fig. 8) to allow the comparison of the laboratory experiments (where water EC ranged between 3 and 6 $\textrm{mS}/\textrm{cm}$) with the experimental field cover (where water EC ranged between 2 and 3 $\textrm{mS}/\textrm{cm}$ for the time period studied). The corrected bulk EC $\sigma _{\textrm{corr}}$ is obtained using (e.g.,^60,110^):

3 $$\begin{aligned} \sigma _{\textrm{corr}} = \sigma \cdot \left[ \frac{\sigma _{\mathrm {w~std}}}{\sigma _{\textrm{w}}} \right] \end{aligned}$$

where $\sigma _{\textrm{w}}$ is the pore water EC normalized at $25~^{\circ }\textrm{C}$ estimated from the hydrogeological sensor measurements. For all experiments, a reference value of $\sigma _{\mathrm {w~std}}=4~\textrm{mS}/\textrm{cm}$ is chosen. Here again, the pore water EC measured by the sensors is assumed to be laterally constant across all the imaged domain and the vertical profiles of pore water EC measured by the sensors are horizontally extrapolated.

#### Petrophysical relationships

The corrected bulk EC values obtained from the inversion are then compared with VWC measurements at the same location. As discussed by^22^, several petrophysical relationships have been successfully applied for mine tailings to link bulk EC and VWC, such as Archie model, generalized Archie’s Law or Waxman-Smits models (e.g.,^45,60,111,112^). In this study, the three petrophysical models have performed equally to link bulk EC and saturation $S_{\textrm{w}}$ and the simplest model (Archie’s Law) is then selected, despite the small grain size and the presence of sulfide in the tailings ($\simeq 1.22~\%$). As a result, both the contributions from the solid grains conduction and the surface conduction, which might occur at the interface between tailings grains and the pore interstitial water, are neglected in this study. This choice could be supported by the high conductivity of the pore interstitial water, which is assumed to be the main contribution of the bulk electrical conductivity of the tailings. Archie model in unsaturated conditions is generally expressed by^113,114^:

4 $$\begin{aligned} \sigma = \phi ^{m} \cdot S_{\textrm{w}}^n \cdot \sigma _{\textrm{w}} \end{aligned}$$

In this model, $\sigma _{\textrm{w}}$ is the pore fluid EC expressed in $\textrm{mS}/\textrm{cm}$, $\phi$ is the porosity (-) such as VWC $= \phi \cdot S_{\textrm{w}}$, and *m* and *n* are two unitless parameters, commonly referred to as the cementation exponent^115,116^ and the saturation exponent, respectively. An optimized petrophysical model is obtained for each experiment by fitting the Archie parameters *m* and *n* (-) to minimize the root mean square error (RMSE) between the inverted bulk EC and the bulk EC predicted by the petrophysical model for a given measurement of VWC^87^. Moreover, the RMSE, the bias and the precision of ERT-predicted VWC are calculated to assess the accuracy of VWC estimations at the different scales studied^117^.

## Results

### Scale 1: electrical resistivity tempe cell

**Figure 9:**
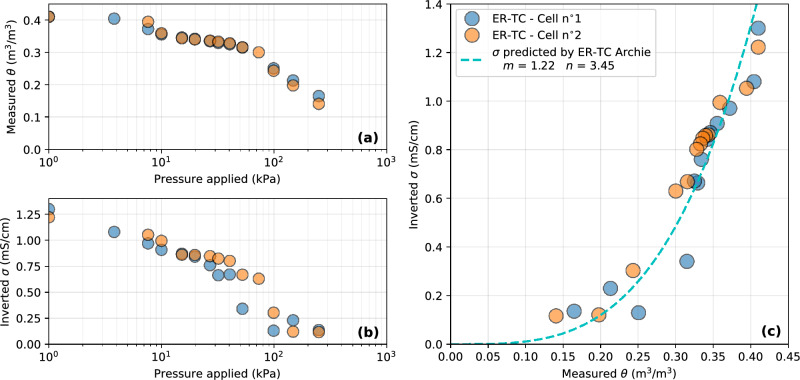
Results from the monitoring of the Electrical Resistivity Tempe Cells (scale 1). Evolution of **(a)** measured VWC and **(b)** inverted bulk EC in the tailings for several pressure steps. **(c)** Relationship between VWC and bulk EC in the ER-TC.

Figure 9 presents the results obtained from the hydrogeophysical measurements in two identical ER-TCs during the drainage of the tailings. The temperature in the cells remained nearly constant at the laboratory temperature ($\simeq ~25~^{\circ }\textrm{C}$) and water outlet sampling allowed to monitor pore water EC over time (between $3.5~\textrm{mS}/\textrm{cm}$ and $4.5~\textrm{mS}/\textrm{cm}$). 14 different pressure steps were applied to the top of the cells, ranging from $1~\textrm{kPa}$ to $250~\textrm{kPa}$. The increase in pressure caused a decrease in VWC from $0.4~\textrm{m}^3/\textrm{m}^3$ to $0.15~\textrm{m}^3/\textrm{m}^3$. The increase in pressure is also associated with a decrease in inverted bulk EC, from $1.3~\textrm{mS}/\textrm{cm}$ at full saturation to a minimum of $0.1~\textrm{mS}/\textrm{cm}$ at maximum pressure. Although the inverted bulk EC distribution was slightly noisier than VWC, especially for Cell n$^{\circ }$1 (blue dots) and at higher pressures, the two parameters seem to be strongly correlated. The evolution of VWC and inverted bulk EC seems consistent with the air entry value of these tailings around 30kPa^78,118^. As expected, bulk EC in the tailings was maximal at high VWC and decreased progressively for lower VWC. The *m* and *n* parameters of an Archie model were optimized to fit the distribution of inverted bulk EC and measured VWC $~\phi =0.4$ and using a reference temperature $T = 25~^{\circ }\textrm{C}$ and a reference pore water EC $\sigma _{\textrm{w}} = 4~\textrm{mS}/\textrm{cm}$. The values $m=1.22$ and $n=3.45$ performed the best with a RMSE value of $0.12~\textrm{mS}/\textrm{cm}$.

### Scale 2: laboratory bucket

**Figure 10:**
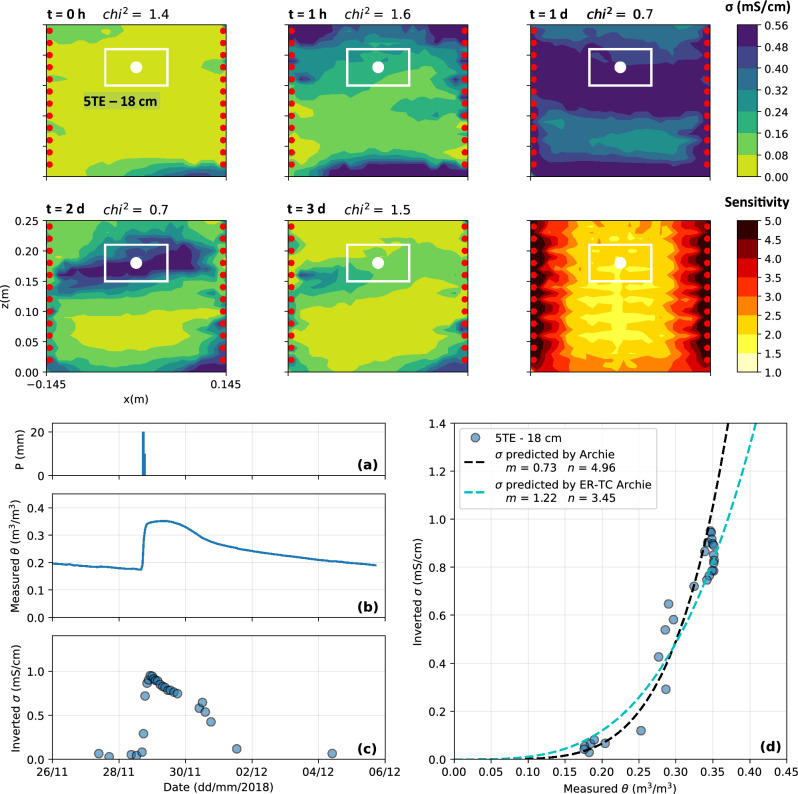
Top panel: 2D slices of the 3D inverted bulk EC distribution and sensitivity for the laboratory bucket (scale **S2**) at selected time steps. The VWC sensor location is indicated by a white dot and the white rectangle corresponds to its volume of investigation, where inverted bulk EC is extracted to be compared with VWC measurements. Bottom panel: evolution of **(a)** artificial precipitations, **(b)** VWC and **(c)** inverted bulk EC in the tailings during the artificial precipitation event and **(d)** petrophysical relationship between VWC and bulk EC in the bucket.

The top panel of Fig. 10 presents the inverted bulk EC distribution in the laboratory bucket at selected time steps, before, during and after an artificial precipitation event of 50 mm. As expected, water infiltration increased bulk EC in the tailings from below $0.1~\textrm{mS}/\textrm{cm}$ to above $0.6~\textrm{mS}/\textrm{cm}$ soon after the infiltration event. Bulk EC increased in the bottom of the bucket a few minutes after the start of the experiment, which suggests that preferential flow occurred from the sides of the bucket. Bulk EC slowly decreased after a few days to come back to initial values after 3 days. Finally, the sensitivity distribution indicates that sensitivity is maximal near electrodes on the sides of the bucket as expected, and decreases toward the center of the bucket, where the 5TE sensor is installed. As shown on the bottom panel of Fig. 10 for one precipitation event, VWC and inverted bulk EC show similar patterns in the tailings. A sharp increase in VWC and bulk EC was observed following each $50~\textrm{mm}$ simulated precipitation event and a slower decrease was reported then, which lasted approximately one week after the precipitation event. The temperature in the tailings ranged from 19 to $24~^{\circ }\textrm{C}$ throughout the experiment, whereas the pore water EC remained mostly constant at $3.5~\textrm{mS}/\textrm{cm}$. The petrophysical model obtained for this experiment (black dashed line on Fig. 10d)) with the parameters $m=0.73$ and $n=4.96$ fits the hydrogeophysical datasets with a RMSE value of $0.15~\textrm{mS}/\textrm{cm}$, and is similar to the ER-TC Archie model (blue dashed line), especially for VWC values lower than $0.35~\textrm{m}^3/\textrm{m}^3$.

### Scale 3: laboratory column

**Figure 11:**
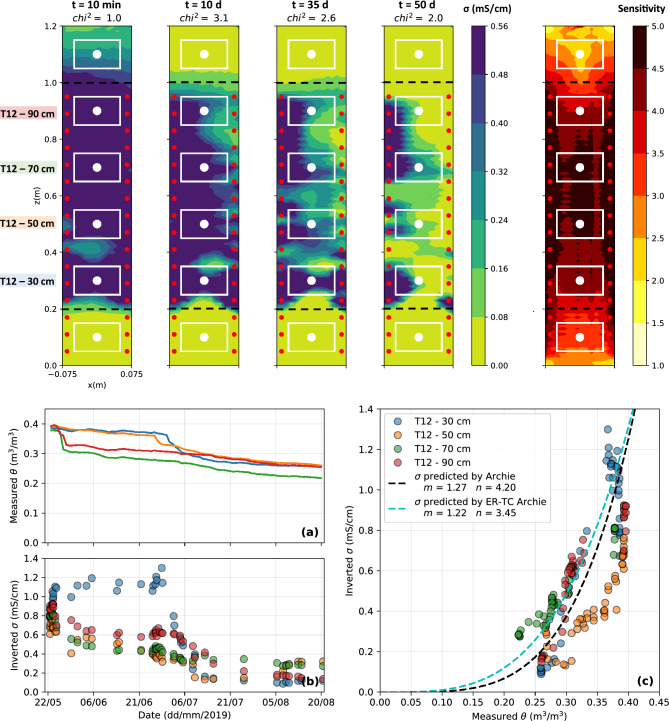
Top panel: 2D slices of the 3D inverted bulk EC distribution and sensitivity for the laboratory column (scale **S3**) at selected time steps. The VWC sensor location is indicated by white dots and the white rectangles correspond to their volumes of investigation. Bottom panel: evolution of **(a)** VWC and **(b)** inverted bulk EC in the tailings during the artificial precipitation event and **(c)** petrophysical relationship between VWC and bulk EC in the bucket.

The top panel of Fig. 11 presents 2D slices of the inverted bulk EC in the laboratory column at different time steps. Bulk EC was high in the saturated tailings at the beginning of the experiment ($>0.6~\textrm{mS}/\textrm{cm}$) and dropped to below $0.1~\textrm{mS}/\textrm{cm}$ after 50 days of drainage and evaporation, especially at the top of the tailings layer where the lowest values of bulk EC were observed. Globally, the waste rocks layers at the bottom and the top of the column remained highly resistive throughout the experiment ($<0.02~\mathrm {mS/cm}$). The sensitivity was maximal in the tailings layer and around electrodes, while lower sensitivities were observed in the waste rocks. As shown by the bottom panel of Fig. 11, VWC ranged between 0.38 and $0.40~\textrm{m}^3/\textrm{m}^3$ when the materials were initially saturated. Two sensors on the top of the tailings (in red and green on Fig. 11**a)** evidenced a sharp drop in VWC down to $0.33~\textrm{m}^3/\textrm{m}^3$, approximately five days after the beginning of the experiment, whereas the two sensors in the bottom of the tailings layer (in orange and blue) remained near saturation during approximately 40 days. These sensors evidenced a strong decrease in VWC after 40 days, which corresponds to the installation of an evaporation fan. The same trends were observed from inverted bulk EC variations, especially at the bottom of the tailings, where inverted bulk EC remained near its initial value ($0.8~\textrm{mS}/\textrm{cm}$) until day 40 and then dropped to $0.1~\textrm{mS}/\textrm{cm}$. The temporal variations of VWC and inverted bulk EC values are mostly consistent, except at $z=50~\text {cm}$ (orange sensor), where the bulk EC seems to decrease more rapidly than the measured VWC. Finally, the temperature and the pore water EC within the tailings ranged between 21 and $27~^{\circ }\textrm{C}$ and between 3.5 and 5.5 $\textrm{mS}/\textrm{cm}$, respectively. The optimized Archie model (black dashed line on Fig. 11**c)**) with the parameters $m=1.27$ and $n=4.20$ fits the data with a RMSE of $0.24~\textrm{mS}/\textrm{cm}$.

### Scale 4: field cover at local scale

**Figure 12:**
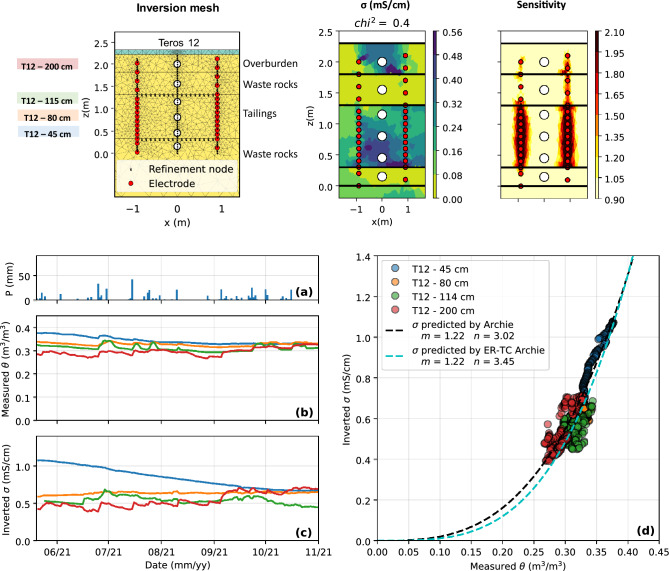
Top panel: inversion mesh, 2D inverted bulk EC distribution and sensitivity in the experimental field cover at local scale (scale **S4**) for a representative time step. The VWC sensors are indicated by white dots and electrodes correspond to red dots. Bottom panel: evolution of **(a)** precipitations, **(b)** VWC and **(c)** inverted bulk EC in the tailings from May to November 2021 and **(d)** relationship between VWC and bulk EC in the experimental CCBE cover at local scale.

The top panel of Fig. 12 presents the 2D inversion mesh defined with pyGIMLi/BERT and the distribution of inverted bulk EC and sensitivity in the CCBE at local scale. The addition of refinement nodes, sensors and horizontal layers allowed to refine the mesh where needed. The inversion of field ERT data shows that the waste rocks are highly resistive ($\sigma <0.02~\text {mS/cm}$), whereas the tailings and the overburden materials are more conductive, with bulk EC ranging from 0.3 to $0.6~\text {mS/cm}$ (before temperature and pore water EC corrections). A slight increase in bulk EC with depth is also observed in the tailings layer. As expected, the sensitivity is highest near the electrodes and decreases sharply in the rest of the imaging domain. Higher sensitivities are observed in the tailings layer, which is consistent with the smaller electrode spacing and the higher bulk EC reported in the tailings.

The bottom panel of Fig. 12 shows the evolution of VWC and bulk EC in the CCBE at local scale from May to November 2021 at local and pilot scale. VWC remained above $0.3~ \textrm{m}^3/\textrm{m}^3$ in the tailings (blue, orange and green lines), which suggests that the capillary barrier effects at the bottom and at the top of the moisture-retaining layer are effective. Every significant precipitation event ($>20~\mathrm {mm/day}$) is followed by an increase in VWC, particularly marked at the top of the tailings layer (green line), where the VWC is always lower than in the rest of the moisture-retaining layer, and in the overburden layer at $z=200~\text {cm}$ (red line). Similar trends can be observed from the variations of inverted bulk EC at both scales, which is generally lower at the top of the tailings layer. A slow but steady decrease of both VWC and inverted bulk EC is observed at the bottom of the tailings layer, as well as a lower response to precipitations event. Similarly, both water content and bulk EC are lower at the top of the cover (green and red sensors) than in the rest of the moisture-retaining layer and exhibit greater temporal variations. Teros 12 sensors also monitored the temperature and pore water EC in the CCBE over time. In contrast to laboratory experiments, strong variations of temperature from $5~^{\circ }\textrm{C}$ to $\simeq 24~^{\circ }\textrm{C}$ were observed from May to November 2021. The pore water EC remained fairly constant in the tailings layer, with values ranging from 2 to 3 $\mathrm {mS/cm}$ throughout the monitoring period. The lower pore water EC values observed in the field (when compared to the laboratory experiments) are likely due to the mixing of initial interstitial pore water ($\sigma _{{\rm w}} \simeq 5.0 ~\hbox{mS/cm}$) and precipitations ($\sigma _{{\rm w}} < 0.1 ~\hbox{mS/cm}$), which has been occurring since the construction of the covers in Fall 2019. Figure 12d demonstrates that the evolution of VWC and bulk EC are consistent in the CCBE at local scale, since the optimized Archie model ($m=1.22$ and $n=3.02$) fits the hydrogeophysical data with a RMSE of $0.08~\text {mS/cm}$. It should be noted that the values from the sensor located in the overburden material (red sensor) were not considered for the fit of the petrophysical model or the RMSE calculations.

### Scale 5: field cover at pilot scale

**Figure 13:**
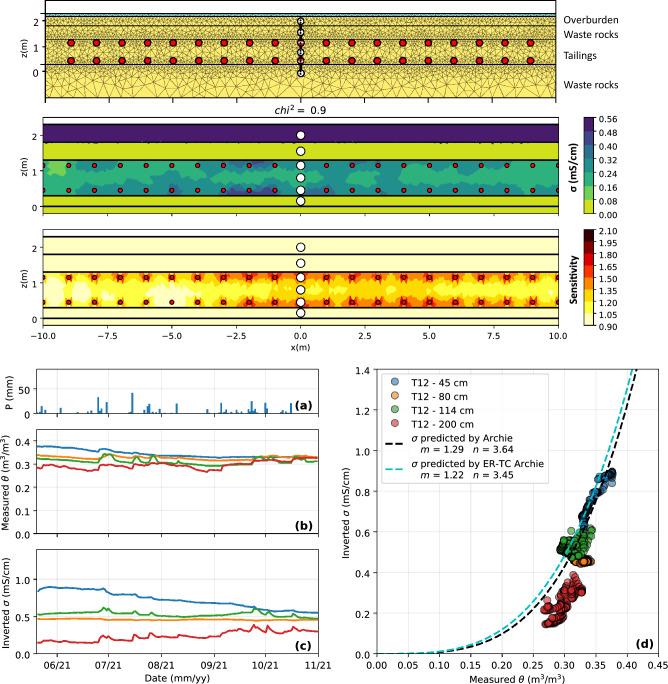
Top panel: inversion mesh, 2D inverted bulk EC distribution and sensitivity in the experimental field cover at pilot scale (scale **S5**) for a representative time step. The VWC sensors are indicated by white dots and electrodes correspond to red dots. Bottom panel: evolution of **(a)** precipitations, **(b)** VWC and **(c)** inverted bulk EC in the tailings from May to November 2021 and **(d)** relationship between VWC and bulk EC in the experimental CCBE cover at pilot scale.

The top panel of Fig. 13 shows the inversion mesh used for TL-ERT inversion of field data and the distribution of inverted bulk EC and sensitivity for the 23 m-long CCBE (scale **S5**). The inverted bulk EC values obtained at pilot scale are consistent with the results presented above at local scale. While inversion sensitivity is high near the electrodes in the tailings layer, poor sensitivity is observed in the overburden and waste rocks layers, as well as in the middle of the tailings layer ($z=80~\text {cm}$) and near dysfunctional electrodes. As shown by the bottom panel of Fig. 13, the temporal evolution of VWC and inverted bulk EC are consistent, and bulk EC is particularly well reconstructed by inversion in the tailings layer at the green and blue sensor locations (top and bottom of the tailings). On the contrary, bulk EC does not match well VWC measurements in the middle of the tailings layer (orange sensor on Fig. 13b) and in the overburden (red sensor), which is likely to be due to the lack of sensitivity at these locations. Globally, the optimized petrophysical Archie model ($m=1.29$ and $n=3.64$) fits the hydrogeophysical dataset with a RMSE value of $0.13~\text {mS/cm}$ at pilot scale as shown in Fig. 13d.

### Relative importance of temperature and pore water EC corrections at different scales

**Figure 14:**
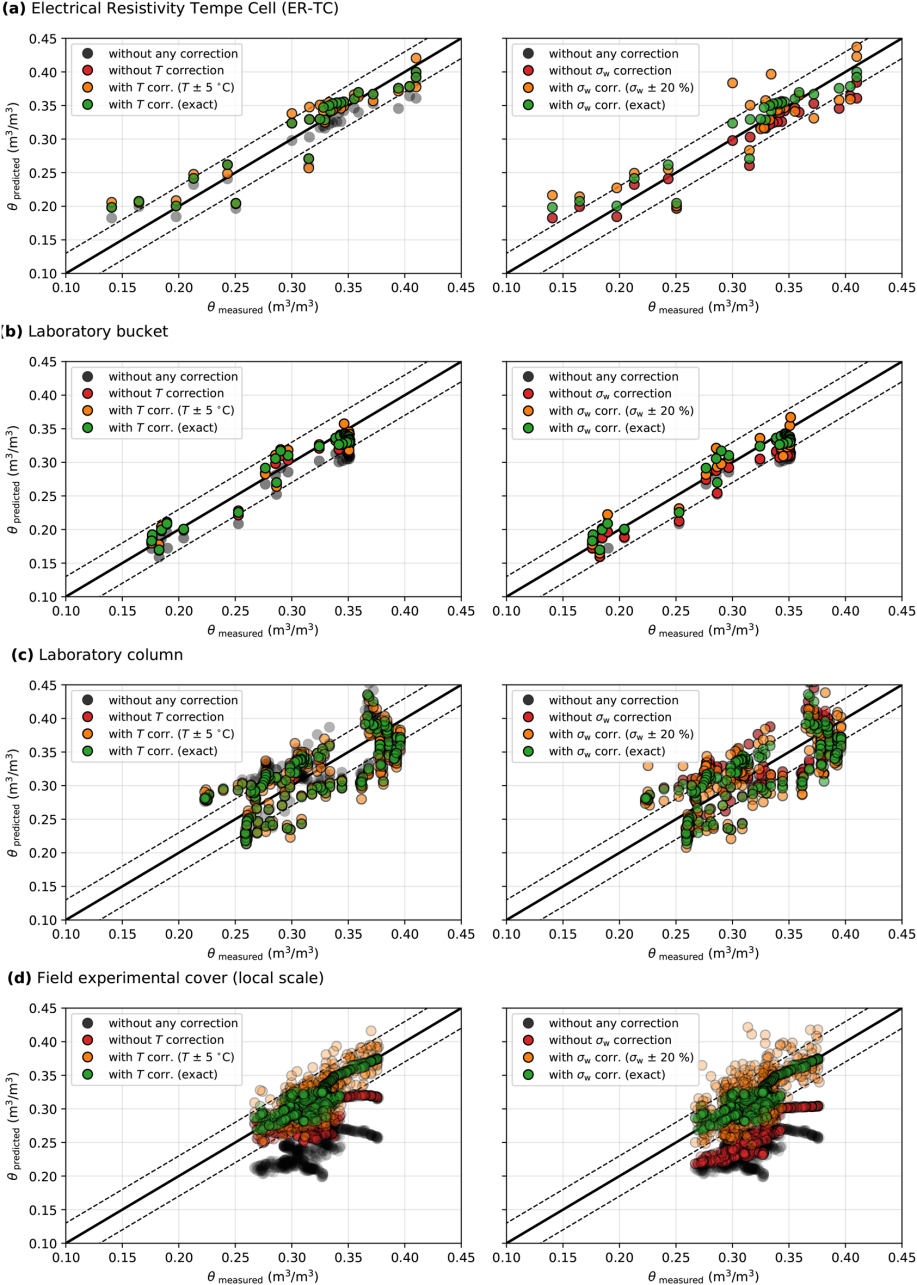
Influence of temperature and pore water EC corrections on the accuracy of ERT-predicted VWC for **(a)** Scale **S1** - Electrical Resistivity Tempe Cell, **(b)** Scale **S2** - laboratory bucket, **(c)** Scale **S3** - Laboratory column and **(d)** Scale **S4** - Field cover at local scale (similar results for scale **S5** not shown here). The 1:1 line indicates a perfect match between ERT-predicted and measured VWC and the black dashed lines indicate an error of $\pm 0.03~\textrm{m}^3/\textrm{m}^3$.

Figure 14 compares the influence of the temperature correction (left panel) and the pore water EC correction (right panel) applied to the inverted bulk EC to predict VWC for scales S1 to S4. The results of scale S5 are very similar to the ones of scale S4 and are not shown here for simplicity. Measured and ERT-predicted VWC are compared (i) when no corrections are applied, (ii) when the temperature correction is not applied, (iii) when the temperature correction is applied using noisy temperature datasets (random noise of $\pm 5~^{\circ }\textrm{C}$) and (iv) when the temperature correction is applied using exact temperature. The same approach is followed for the pore water EC correction using a noise of $\pm 20~\%$ of the measured pore water EC.

For all experiments, most of the ERT-predicted VWC values lie between the two black dashed lines when the two corrections are properly applied, which indicates that the overall accuracy of ERT-predicted VWC is near $0.03~\textrm{m}^3/\textrm{m}^3$. The accuracy of VWC predictions decreases when the temperature or/and the pore water EC corrections are not applied, particularly for the field experiments (Scale **S4** and **S5**). At scale **S4** in particular, the RMSE between ERT-predicted and measured VWC is (i) $0.077~\textrm{m}^3/\textrm{m}^3$ when no corrections are applied, (ii) $0.038~\textrm{m}^3/\textrm{m}^3$ when the temperature correction is not applied, (iii) $0.053~\textrm{m}^3/\textrm{m}^3$ when the pore water EC correction is not applied, and (iv) $0.013~\textrm{m}^3/\textrm{m}^3$ when the two corrections are properly applied. Similarly, the precision of ERT-predicted VWC decreases when noisy temperature and pore water EC are used, particularly for the field experiments (Scales **S4** and **S5**). Indeed, the precision of ERT-predicted VWC in the field CCBE is two times lower when a $\pm 5~^{\circ }\textrm{C}$ noise is randomly applied to the temperature dataset (as opposed to an exact temperature). The same observation is made when a $\pm 20~\%$ noise is randomly applied to the pore water EC dataset. The accuracy of ERT-predicted VWC is less affected by the temperature and pore-water EC corrections for laboratory experiments, which can be explained by smaller variations of temperature and pore water EC.

### Multi-scale comparison of petrophysical models and ERT-predicted VWC accuracy

**Figure 15:**
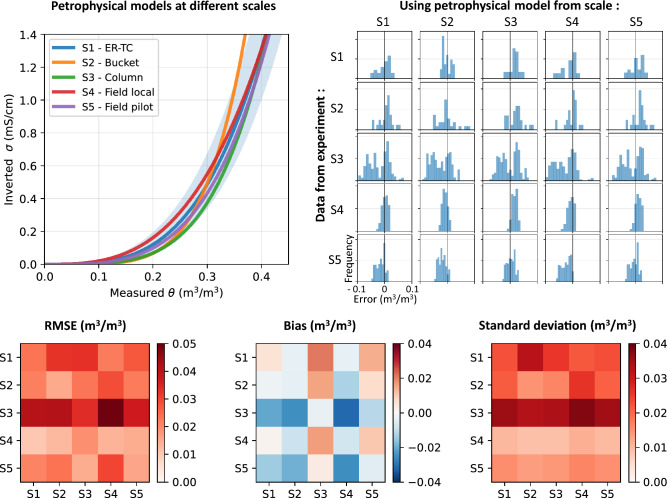
Assessment of the scale influence on petrophysical models and on the accuracy of ERT-predicted VWC. Top left - Comparison of the petrophysical models calibrated using hydrogeophysical datasets from scales **S1** to **S5**. Top right - Histograms of the error between measured and ERT-predicted VWC using data and petrophysical models from different scales. Bottom panel - RMSE, bias and standard deviation of VWC prediction errors using ERT data and petrophysical models from different scales.

The top left panel of Fig. 15 compares the petrophysical models calibrated using hydrogeophysical datasets at various scales. All petrophysical models are similar to each other since the models from scales **S2** to **S5** are encompassed within the area defined around the ER-TC model in shaded blue (scale **S1**), considering an uncertainty of $\pm 0.03~\textrm{m}^3/\textrm{m}^3$. The histograms of errors between measured and ERT-predicted VWC shown in the top right panel of Fig. 15 assess the accuracy of ERT-predicted VWC. As expected, the lowest RMSE and bias values are observed for the diagonal elements, i.e., when the same hydrogeophysical datasets are used for the calibration of petrophysical models and the prediction of VWC using ERT for each scale. However, it can be observed that RMSE and bias values remain below $0.03~\textrm{m}^3/\textrm{m}^3$ and $\pm 0.01~\textrm{m}^3/\textrm{m}^3$, respectively, when a petrophysical model defined at a specific scale is used to predict VWC at a different scale (i.e., non-diagonal elements). For instance, the left column of the matrix indicates that RMSE and bias of ERT-predicted VWC using the ER-TC petrophysical model are below $0.04~\textrm{m}^3/\textrm{m}^3$ and $\pm 0.02~\textrm{m}^3/\textrm{m}^3$, respectively, for the different scales.

## Discussion

### Overall accuracy of ERT-predicted VWC

This study compares VWC measured using hydrogeological sensors and ERT-predicted VWC using Archie petrophysical models at different scales and for different hydrogeological conditions (drainage, wetting and natural precipitations). Overall, the reliability of ERT-predicted VWC is similar to the accuracy of moisture content sensors at the different scales studied. Indeed, both low bias and high precision are achieved (below $0.01~\textrm{m}^3/\textrm{m}^3$ in absolute value and below $0.03~\textrm{m}^3/\textrm{m}^3$, respectively). In addition, RMSE values do not exceed $0.035~\textrm{m}^3/\textrm{m}^3$ at the different scales investigated in this study. As a result, VWC dynamics are well imaged using TL-ERT at the different scales, both during mine tailings drying, wetting and drying cycles and for field meteorological conditions. The latter highlights the strong potential that TL-ERT could represent as a monitoring approach used in combination with point moisture sensors to increase the investigation volume of future monitoring programs in reclamation covers (e.g.,^45,74^), as this is the case for monitoring landslide, permafrost, geotechnics or $\textrm{CO}_2$ storage, for example^11,22,119^.

The accuracy of ERT-derived VWC reported in this study is consistent with previous studies for other fields of applications, including hydrogeological studies^52,120^, waste landfills imaging^121^, root zone dynamics monitoring^57,122^ and irrigation studies^2,56^. Indeed, most of these studies reported RMSE values ranging between 0.01 and $0.04~\textrm{m}^3/\textrm{m}^3$ (e.g.,^59,123,124^). Generally, local moisture sensors (e.g., TDR sensors or capacitance probes), neutron probes, numerical modelling or direct oven-weighting methods are used to measure VWC and evaluate the accuracy of ERT-predicted VWC (e.g.,^52,53,56,57,120,125^). Bias and precision of ERT-predicted VWC reported in some studies are also consistent with the results of this study (e.g.,^126–128^).

Some authors report higher accuracy for ERT-predicted VWC in wet conditions (in comparison with dryer conditions)^52,57,61^. This observation is consistent with the results obtained at the sample scale (scale **S1** - electrical resistivity Tempe cell, see Section “Scale 1: electrical resistivity tempe cell”). The latter could be explained in part by a better electrical contact between the electrodes and the surrounding medium at higher saturation, which in turn improves the signal-to-noise and reduces measurement errors although in general, more conductive conditions reduce the transfer resistance and reduce signal-to-noise^61^. Nonetheless, it should be noted that for field applications (scales **S4** and **S5**), an accuracy of $\pm 0.03~\textrm{m}^3/\textrm{m}^3$ would correspond to an error of at least $\pm 30\%$, given that a maximum amplitude of variation of $0.10~\textrm{m}^3/\textrm{m}^3$ was reported. For these scales in particular, the small variations of VWC during the monitoring period did not allow to assess the accuracy of TL-ERT to recover strong spatio-temporal dynamics of VWC. The choice of the cascade inversion framework could also have hindered the reconstruction of quick dynamics of VWC, especially for the infiltration events in the bucket (scale **S2**), thus reducing the accuracy of ERT-predicted VWC. In this regard, it could have been relevant to include 4D effects through a full time-lapse inversion framework (e.g.,^129–131^). Finally, although the experiments described in this study did not allow to assess the impact of hysteresis on VWC and on the relationship between VWC and bulk EC, this could have an effect on the accuracy on ERT-predicted VWC^132–134^.

### Importance of temperature and pore water EC corrections for field applications

This study highlights the importance of temperature and pore water EC corrections for accurate predictions of VWC in tailings using ERT, particularly for field applications. Indeed, the ERT-predicted VWC is systematically underestimated when no corrections are applied for field experiments (scales **S4** and **S5**), with a corresponding tenfold reduction in accuracy. Such underestimation is consistent with the lower temperature and the lower pore water EC reported in the field in comparison with laboratory experiments. A decrease of $10~^{\circ }\textrm{C}$ in temperature from $20~^{\circ }\textrm{C}$ to $10~^{\circ }\textrm{C}$ causes a decrease greater than 20% in bulk EC (e.g.,^106,135^). As noted by^136^, the temperature correction is all the more important for geoelectrical monitoring surveys with large temporal changes in temperature (e.g., for field experiments with daily or seasonal variations of subsurface temperature)^56,109^. The latter is especially true at Canadian Malartic mine, since extreme air temperature changes are frequently observed between Winter ($-30~^{\circ }\textrm{C}$) and Summer ($30~^{\circ }\textrm{C}$)^137^ or in similar contexts (e.g., permafrost monitoring surveys^138^). Similarly, this study highlights that pore water EC must be taken into account to accurately predict VWC from ERT datasets since bulk EC and pore water EC are proportional in the Archie petrophysical model. For instance, the lower pore water EC reported for the field cover must be considered in order to avoid underestimation of VWC in the field. Here again, the pore water EC correction is particularly important in the context of mining wastes, given (i) the strong pore water EC contrasts that are generally observed between clean water and water in contact with tailings and waste rocks, whether or not affected by contaminated drainage and (ii) the strong temporal variations of pore water EC in mining wastes (e.g.,^45,139,140^).

For the laboratory experiments, the temperature and pore water EC corrections have a smaller influence on the RMSE between the measured and ERT-predicted VWC. The latter can be explained by smaller temporal variations of temperature throughout the experiment under controlled conditions and by a smaller difference between the actual temperature and the reference value used for the temperature correction (the same observation can be done for the influence of pore water EC correction in the laboratory)^55^. As a result, this study shows that considering the temperature and the pore water EC is necessary to up-scale laboratory petrophysical relationships at the field scale in order to accurately predict VWC from TL-ERT monitoring. This further highlights the importance of using complementary hydrogeological sensors for field surveys to locally monitor temperature, pore water EC and VWC, which would be used both as input and validation data for processing TL-ERT datasets^1,18^. As noted by^141^, the key question for future hydrogeophysical monitoring programs would then be how to select the critical number of sensors and how to optimally deploy them in the field (e.g.,^142,143^). In particular, strategies should be investigated to tackle the impact of pore water EC at large scales, especially in media where strong spatial variability of pore water EC is expected. This aspect yields to a limitation of this study; the temperature and the pore water EC measured with sensors have been considered laterally constant. This assumption is quite strong and many sites may not respect it, especially for heterogeneous media and at larger scales than the ones investigated in this study (maximum of 24 m). In this regard, the error in temperature and/or pore water EC which might be due to lateral variations of these parameters could yield lower accuracy in the ERT-predicted VWC at larger scales. A similar observation could be made for the effect of spatially-varying compaction of the material, given the role of the porosity in the petrophysical models. As a result, the quantitative approach described in this study may not be applicable if strong spatial variations of porosity, temperature or pore water EC are expected. Finally, as discussed by^22^ and^21^, it might be challenging and costly to install many calibration sensors, especially if the installation of these sensors modifies the surrounding medium. For instance, it is suspected that a lower compaction was achieved around some sensors in the tailings (especially for scales **S2** and **S3**), which could have led to a systematic bias in the comparison between VWC and bulk EC.

### Validity of Archie model for mine tailings

In this study, the Archie petrophysical model seems appropriate given the small RMSE obtained between the inverted and predicted bulk EC ($\epsilon _{\sigma } < 0.25~\mathrm {mS/cm}$) and between measured and ERT-predicted VWC ($\epsilon _{\theta } < 0.04~\textrm{m}^3/\textrm{m}^3$). The cementation exponent *m* and the saturation exponent *n* obtained for this study range from 0.7 to 1.3 and from 3.0 to 5.0 respectively. It is interesting to note that different couples of *m* and *n* parameters yield similar petrophysical models, which highlights the non-uniqueness of such optimization approaches, as discussed recently by^87^. As reported by^45^ and^22^, few studies have investigated the applicability of petrophysical relationships between bulk EC and VWC for mine tailings specifically, although this has been done for other fields of applications (e.g.,^46,93,144^). Nonetheless, the Archie parameters obtained in this study are consistent with the values reported in the literature for tailings. For example^112^, obtain a $r^2$ value of 0.90 between saturation and bulk EC in tailings with a cementation exponent $m=1.3$ and a saturation exponent $n=3.8$. Similarly^60^, obtain $(m=2.7,n=3.0)$ in mine tailings.

The values of the cementation exponent *m* correspond well to the typical values reported in the literature for unconsolidated media, generally between 1.1 and 1.3, whereas *m* values between 1.7 and 2.1 are generally used for consolidated media^50,51,112^. Interestingly, the values of saturation exponent *n* obtained in this study (between 3.0 and 5.0), and in other ERT studies for tailings do not correspond to the generally assumed value of $n\simeq 2$ for media where the interstitial fluid is water^145,146^. As discussed by^112^ and^128^, such discrepancies are also pointed out by several other studies which consider a larger range for the saturation exponent *n* (between 1.5 and > 10)^147,148^.

Petrophysical models other than the Archie relationship could have been considered in this study to connect bulk EC and VWC in mine tailings. For example^60^, successfully applied Waxman-Smits^149^ and generalized mixing Archie models^63^ to predict VWC in mine tailings from geoelectrical monitoring. An advantage of these models is that they allow to include the contribution of surface conduction, which could play a role in the bulk EC of tailings (e.g.,^150,151^). In this study, however, the high pore water EC observed in the tailings is assumed to make the pore fluid conductivity the predominant contribution of bulk EC, thus rendering both solid matrix conduction and surface conduction negligible^144,152^. Nonetheless, further work could be done at the laboratory scale under controlled conditions using tailings with a lower pore water EC to increase the relative contribution of solid matrix and surface conduction (e.g.,^153^).

Selecting an optimal petrophysical model for tailings is still open to future investigation. Some aspects of the relationship between various physical properties in mine wastes, bulk EC and VWC are still poorly understood, despite recent developments (e.g.,^60,111,154,155^). Future efforts could be devoted to the development of predictive models to assess the relationships between bulk EC and VWC for specific tailings, given their grain size distributions, clay content, mineralogy, ionic content and $\textrm{pH}$ of the interstitial fluid, for example, following the work of recent studies (e.g.,^155–160^. The latter is already commonly done for the prediction of hydrogeological properties from basic geotechnical properties of mining wastes (e.g., prediction of water retention curves^161,162^).

### Scale influence on petrophysical models

This study also demonstrates that the Archie petrophysical model calibrated at sample scale using the electrical resistivity Tempe cell (ER-TC - scale **S1**) turns out to be globally valid at larger scales as shown by the maximal RMSE obtained using the ER-TC Archie model remaining below $0.040~\textrm{m}^3/\textrm{m}^3$ for scales **S2** to **S5**. As a result, it seems appropriate for mine tailings to carry out experiments in the laboratory on small samples to calibrate a petrophysical model and to apply this model on a larger scale, given that the suitable corrections are properly applied. This observation is all the more relevant as most studies using TL-ERT to predict VWC rely on laboratory-based petrophysical relationships to quantify VWC at the field scale, assuming that the petrophysical model obtained at sample scale can be scaled up (e.g.,^9,16,53,61^).

For example^59^, and^163^ review different laboratory apparatus that have been used to characterize the relationship between bulk EC and VWC at sample scale (e.g.,^164–167^). Among the different types of laboratory apparatus developed recently, the most promising are modified from well-documented geotechnical or hydrogeological laboratory apparatus (e.g., permeameter, oedometer, Tempe Cell, pressure plate or triaxial cell) to allow a simultaneous determination of VWC (or other relevant physical parameter) and bulk EC of the sample under controlled conditions (e.g.,^87,168–172^). Another popular approach consists in determining the petrophysical relationships from co-located and simultaneous measurements of bulk EC and VWC directly sampled in the field, under natural conditions^97,122^. To our knowledge, this study constitutes the first attempt to compare the petrophysical relationships derived from sample, laboratory and field scale experiments to assess the accuracy of moisture content predictions.

In particular, the electrical resistivity Tempe cell (ER-TC) proposed and tested in this study has considerable potential for ensuring rapid, flexible, reproducible and accurate determination of petrophysical relationships for fine-grained materials, such as mine tailings. Indeed, Tempe Cells or equivalent pressure extractor cells^79,82^ are widespread and commonly used in most hydrogeological laboratories for the assessment of water retention curves (WRC) (e.g.,^173,174^). The slight modification of the Tempe Cell needed to add several lateral electrodes allows determining simultaneously the WRC and the relationship between bulk EC and VWC under controlled conditions. For instance, the experiments can be conducted for controlled temperature, known pore water EC, known porosity and homogeneous compaction, as well as with a lower risk of lateral and preferential flow. Moreover, the number of different pressure steps allows controlling the number of points used to define the petrophysical relationship, and allows defining the range of pressure studied (i.e., the range of VWC of interest), which might not be the case in the field when only small variations of moisture content could be observed. For example, the small variations of VWC in the tailings layer of the field experimental cover only allows to constraint the in-situ petrophysical relationship for the $0.27 - 0.38~\textrm{m}^3/\textrm{m}^3$ VWC interval. In such cases, it might be difficult to extrapolate the petrophysical relationships outside of the range of in-situ observed VWC, as discussed by Tso, Kuras, and Binley^97^.

Another advantage of the small-scale ER-TC apparatus is the high certainty of bulk EC values reconstructed by the inversion process, as opposed to the inversion results obtained from experiments at larger scales, in the field or at the laboratory. The inversion results from small-scale laboratory apparatus are less subject to resolution issues (i.e., area of the medium poorly constrained inversion^175^), non-uniqueness and artifacts of the inversion^11,19,47^, unsuitable inversion regularization^103^ or uncertainty in electrode location^81,176,177^. In particular, several studies have shown that the loss of sensitivity in ERT inversion has an influence on the petrophysical relationships, making the conversion of bulk EC into VWC (or other parameters) less accurate (e.g.,^178–181^). This is especially true in areas poorly constrained by the inversion, for instance far from electrodes or if larger electrode spacing are used. In this regard, it should be noted that the so-called large-scale field experiments described in this study are still high-resolution applications (e.g., electrode spacing of 1 m), which may not be applicable to monitor entire tailings storage facility as discussed by Dimech et al.^22^. As a result, field-scale applications of TL-ERT would likely be carried out with lower spatial resolution, which may reduce the accuracy of ERT bulk EC images, hence reducing the accuracy of ERT-predicted VWC.

In addition, data quality issues are more likely to occur for field applications as opposed to small-scale laboratory tests since lower signal over noise ratios or high contact resistance between the medium and the electrode may be encountered^92,96,182,183^. Nonetheless, it can be observed from Fig. 9 that the bulk EC obtained from small-scale cells could also be noisy, especially for Cell n$^{\circ }$1 (blue dots) for VWC between 0.20 and 0.33 $\textrm{m}^3/\textrm{m}^3$. This might be attributed, at least in part, by a deterioration of the electrical contact between the electrodes and the tailings, which tend to shrink slightly in the cell during the test. As a result, we recommend to duplicate the testing in laboratory for a given material to ensure proper results, as it is generally done for classical Tempe cells^79^. Finally, some differences in the investigation volume between ERT and point moisture sensors installed on the field could make it difficult to compare hydrogeological and geophysical results^1,30,56^. Future studies using similar small-scale laboratory apparatus could focus on determining the optimal electrode shape, material and spacing to maximize electrical coupling with the tailings, even for dry conditions, while ensuring the electrodes remain small enough to ensure they can be considered as points in the numerical models (see^81,184^). In this regard, it is also important to ensure that the material in the cell is compacted at the same porosity as what is expected in the field.

Finally, the use of small-scale, well-constrained and reproducible laboratory apparatus such as the ER-TC presented in this study should be preferred for the assessment of petrophysical relationships and the future investigation of the following challenging issues for VWC prediction in mine tailings using TL-ERT:

- the contribution of the surface conductivity and anisotropy in mining tailings (e.g.,^60,61^),
- the possible reduction of accuracy due to a loss of precision for larger scale applications on the field^22^,
- the prediction of petrophysical relationships from basic material properties and mineralogy (e.g.,^154,155^),
- the influence of porosity spatio-temporal evolution on ERT-predicted VWC (e.g.,^55,185,186^),
- the hysteresis and other non-stationary aspects of petrophysical relationships (e.g.,^53,134,187^).

## Conclusion

In this study, we assess the scale effect on the accuracy of ERT-predicted VWC in mine tailings. In total, five different experimental setups are used to carry out simultaneous and co-located monitoring of bulk EC using TL-ERT and VWC using hydrogeological sensors. These datasets allow to calibrate Archie petrophysical models at various scales, and assess the accuracy of VWC predictions in mine tailings. The accuracy of ERT-predicted VWC is $\pm 0.03~\textrm{m}^3/\textrm{m}^3$, and the petrophysical models determined at sample-scale in the laboratory remain globally valid at larger scales. Indeed, a similar accuracy is achieved when the petrophysical model determined from the sample-scale electrical resistivity Tempe cell (ER-TC) is applied to field data.

This study also highlights the strong impact that temperature and pore water EC variations can represent for the accuracy of VWC predictions using TL-ERT, especially for field applications. Indeed, a tenfold reduction of VWC accuracy is observed in the absence of suitable corrections in the experimental field cover. Based on these results, we recommend that future TL-ERT monitoring campaigns should be combined with networks of hydrogeological sensors, providing local validation and calibration measurements of VWC, temperature, bulk and pore water EC in tailings.

Finally, this study discusses the advantages of using small-scale, well-controlled and reproducible laboratory apparatus such as the ER-TC for the determination of petrophysical models in mine tailings, given that the samples used are representative of field conditions. In particular, a widespread use of similar sample-scale apparatus is likely to help refine petrophysical models applied to mine tailings, hence improving the accuracy and applicability of TL-ERT for mining waste monitoring across large scales.

### Supplementary Information

Supplementary Information 1.

Supplementary Information 2.

Supplementary Information 3.

## Data Availability

The datasets presented in this study are available at the Mendeley Data repository at doi:10.17632/hnm5sczn45.3, an open-source online data repository hosted with CC BY 4.0 licence^188^.
