# Supplementary material for: A multiscale accuracy assessment of moisture content predictions using time-lapse electrical resistivity tomography in mine tailings
Source: Sci Rep. 2023 Nov 27;13:20922. doi: 10.1038/s41598-023-48100-w (PMC10684595; doi:10.1038/s41598-023-48100-w)
Supplement: Supplementary file 1 — Supplementary Information 1. [file 41598_2023_48100_MOESM1_ESM.pdf]

A multiscale accuracy assessment of moisture content predictions using time-lapse electrical resistivity tomography in mine tailings

Adrien Dimech[a,c,*], Anne Isabelle[b,c], Karine Sylvain[b,c], Chong Liu[a], LiZhen Cheng[a,c], Bruno Bussière[a,c], Michel Chouteau[b,c], Gabriel Fabien-Ouellet[b], Charles Bérubé[b], Paul Wilkinson[d], Philip Meldrum[d] and Jonathan Chambers[d]

[a] *Université du Québec en Abitibi-Témiscamingue (UQAT), Rouyn Noranda, J9X 5E4, Québec, Canada*
[b] *Polytechnique Montréal, Montréal, H3T 1J4, Québec, Canada*
[c] *Research Institute of Mines and Environment (RIME), Québec, Canada,*
[d] *British Geological Survey (BGS), Environmental Science Centre, Keyworth, Nottingham, NG12 5GG, United Kingdom*

ABSTRACT

A. Figures in Appendix

*Corresponding author
adrien.dimech@etsmtl.ca (A. Dimech)
ORCID(s): 0000-0001-6078-4300 (A. Dimech); 0000-0002-8538-1775 (M. Chouteau); 0000-0002-1849-3718 (G. Fabien-Ouellet); 0000-0002-5438-0382 (C. Bérubé); 0000-0002-8135-776X (J. Chambers)

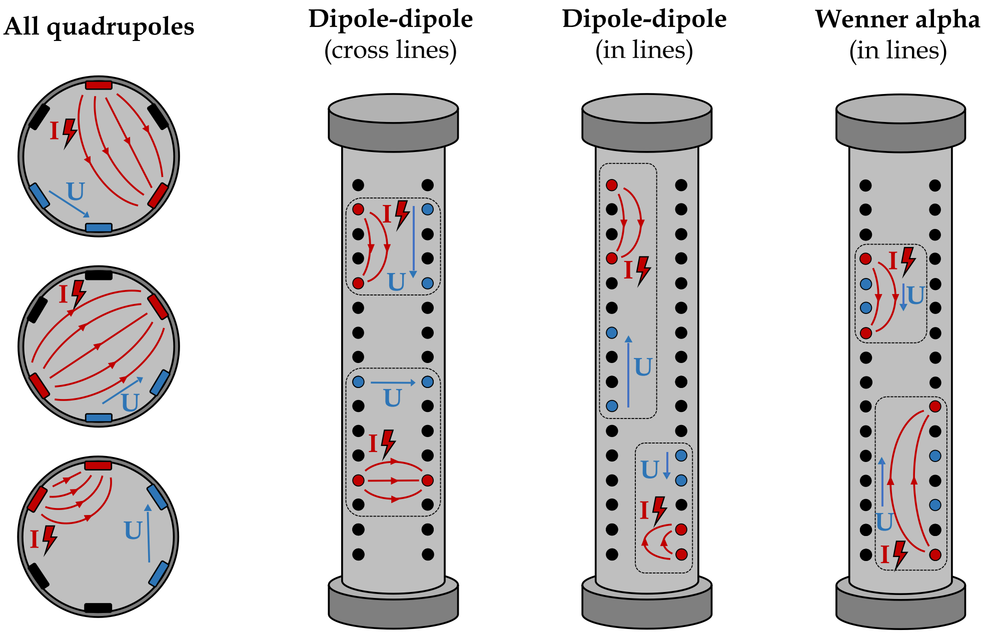

Figure A.1: Illustration of the configurations used to carry out TL-ERT monitoring in the tailings at different scales. Red and blue electrodes represent respectively current and potential electrodes.
