# Supplementary figures and images for: A multiscale accuracy assessment of moisture content predictions using time-lapse electrical resistivity tomography in mine tailings
Source: Sci Rep. 2023 Nov 27;13:20922. doi: 10.1038/s41598-023-48100-w (PMC10684595; doi:10.1038/s41598-023-48100-w)

All quadrupoles

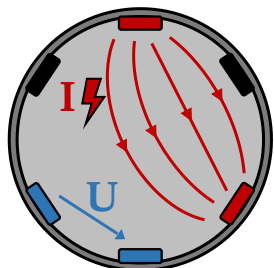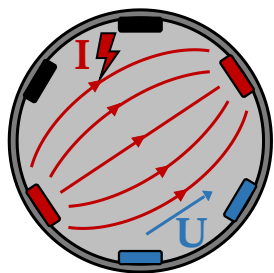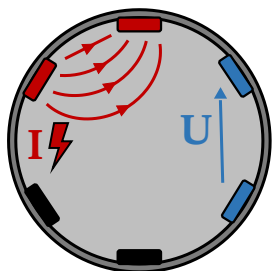

Dipole-dipole  
(cross lines)

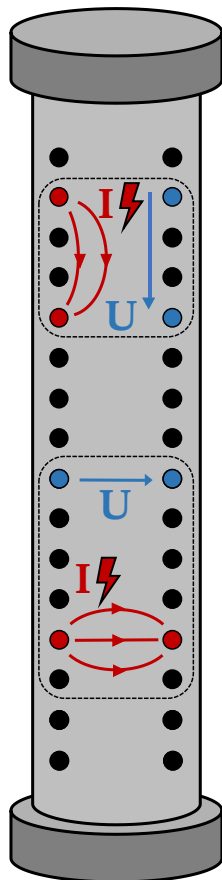

Dipole-dipole  
(in lines)

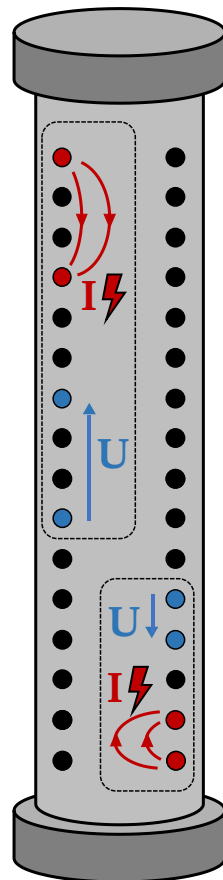

Wenner alpha  
(in lines)

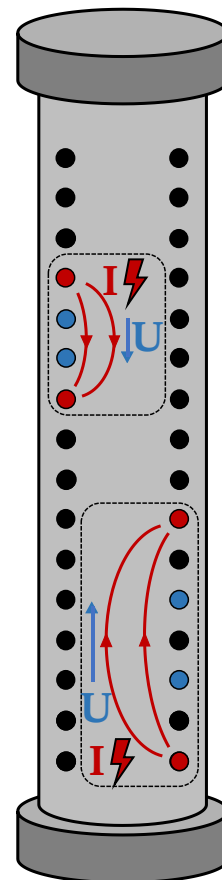

Supplement: Supplementary file 2 — Supplementary Information 2. [file 41598_2023_48100_MOESM2_ESM.zip › figs/Figure_A3.pdf]

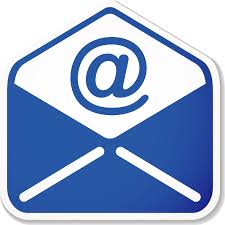

Supplement: Supplementary file 2 — Supplementary Information 2. [file 41598_2023_48100_MOESM2_ESM.zip › thumbnails/cas-email.jpeg]

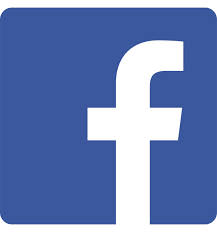

Supplement: Supplementary file 2 — Supplementary Information 2. [file 41598_2023_48100_MOESM2_ESM.zip › thumbnails/cas-facebook.jpeg]

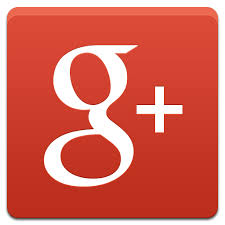

Supplement: Supplementary file 2 — Supplementary Information 2. [file 41598_2023_48100_MOESM2_ESM.zip › thumbnails/cas-gplus.jpeg]

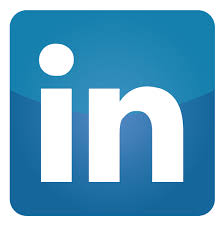

Supplement: Supplementary file 2 — Supplementary Information 2. [file 41598_2023_48100_MOESM2_ESM.zip › thumbnails/cas-linkedin.jpeg]

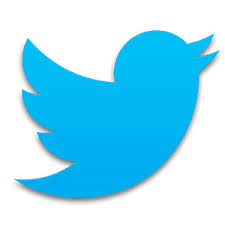

Supplement: Supplementary file 2 — Supplementary Information 2. [file 41598_2023_48100_MOESM2_ESM.zip › thumbnails/cas-twitter.jpeg]

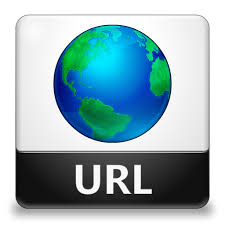

Supplement: Supplementary file 2 — Supplementary Information 2. [file 41598_2023_48100_MOESM2_ESM.zip › thumbnails/cas-url.jpeg]

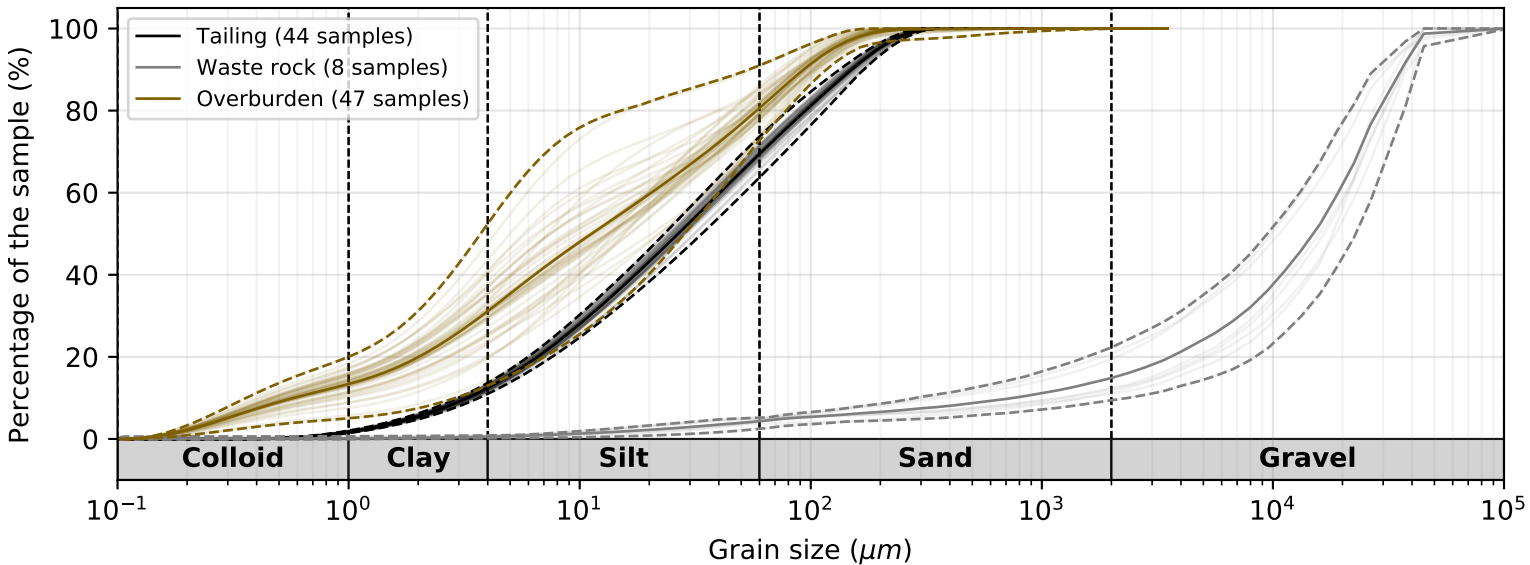

Supplement: Supplementary file 3 — Supplementary Information 3. [file 41598_2023_48100_MOESM3_ESM.zip › figs/Figure_1.pdf]

S1

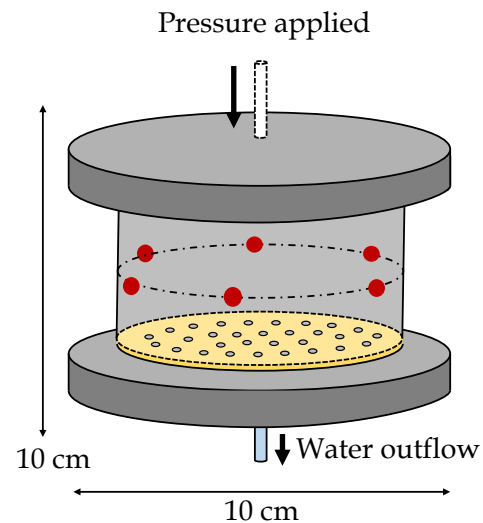

S2

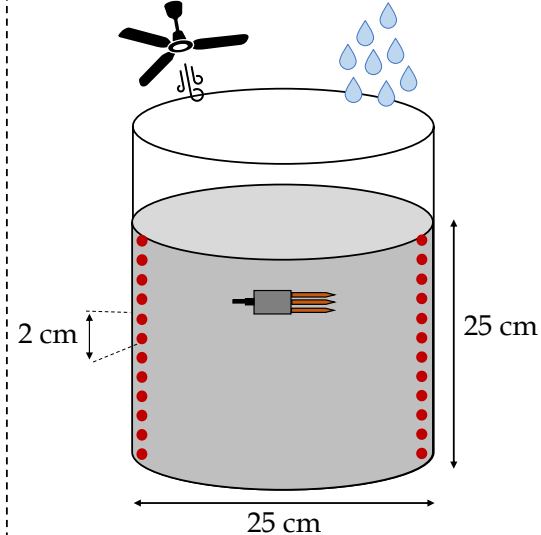

S3

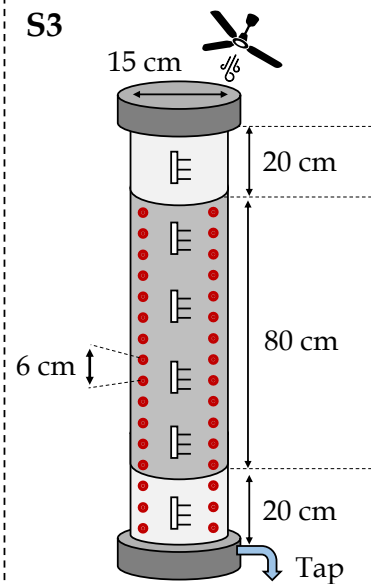

S5

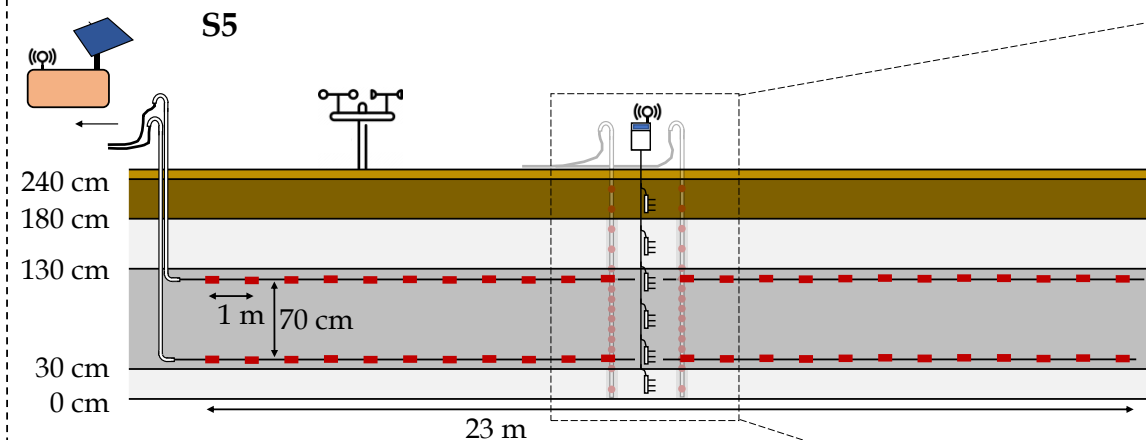

S4

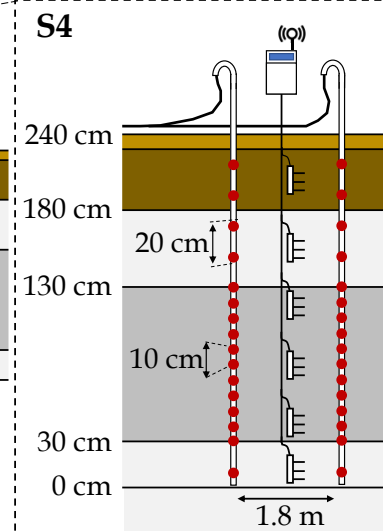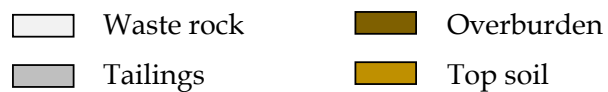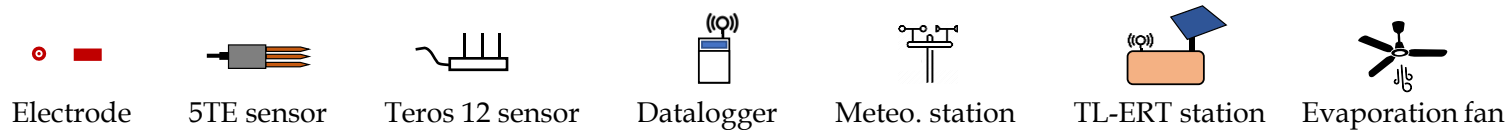

Supplement: Supplementary file 3 — Supplementary Information 3. [file 41598_2023_48100_MOESM3_ESM.zip › figs/Figure_2.pdf]

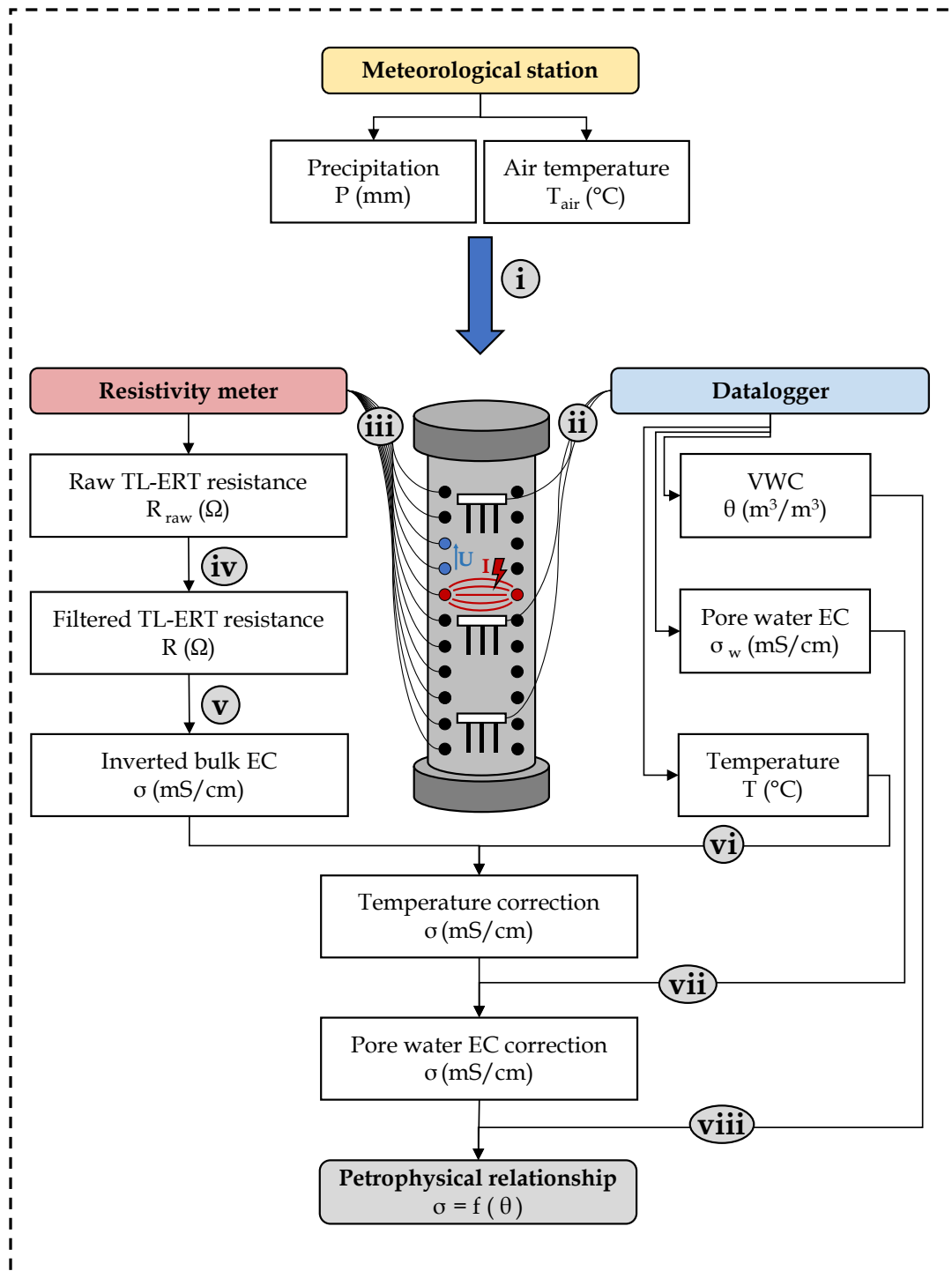

Supplement: Supplementary file 3 — Supplementary Information 3. [file 41598_2023_48100_MOESM3_ESM.zip › figs/Figure_8.pdf]

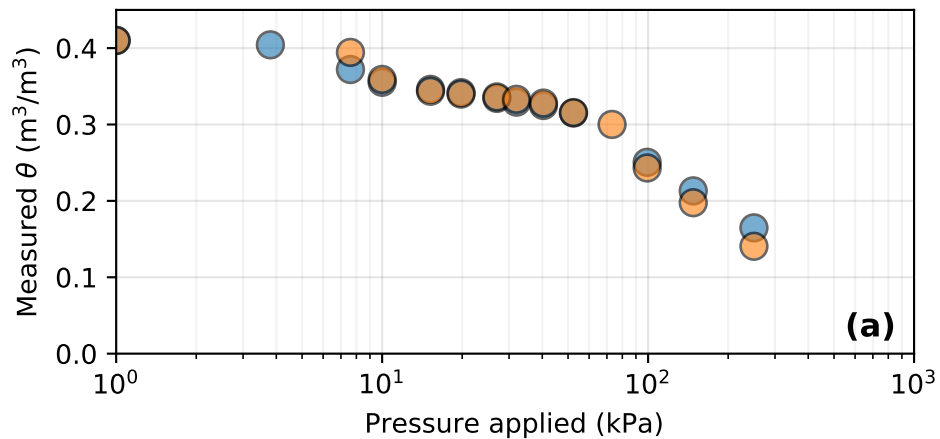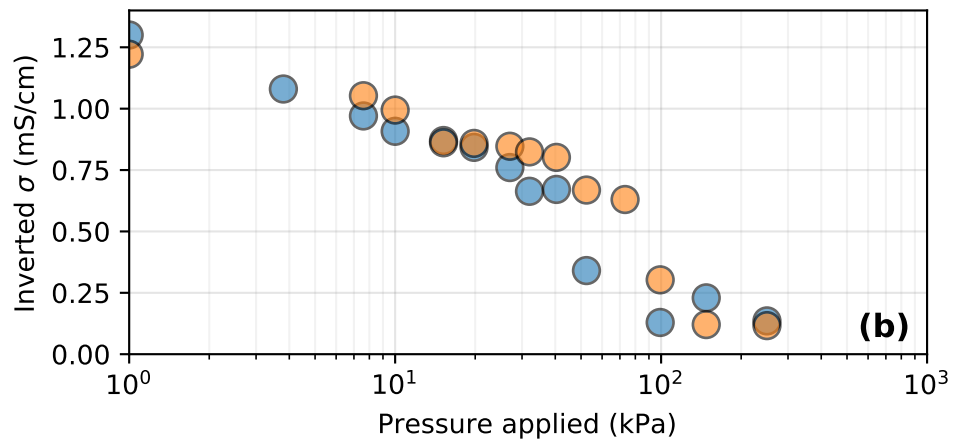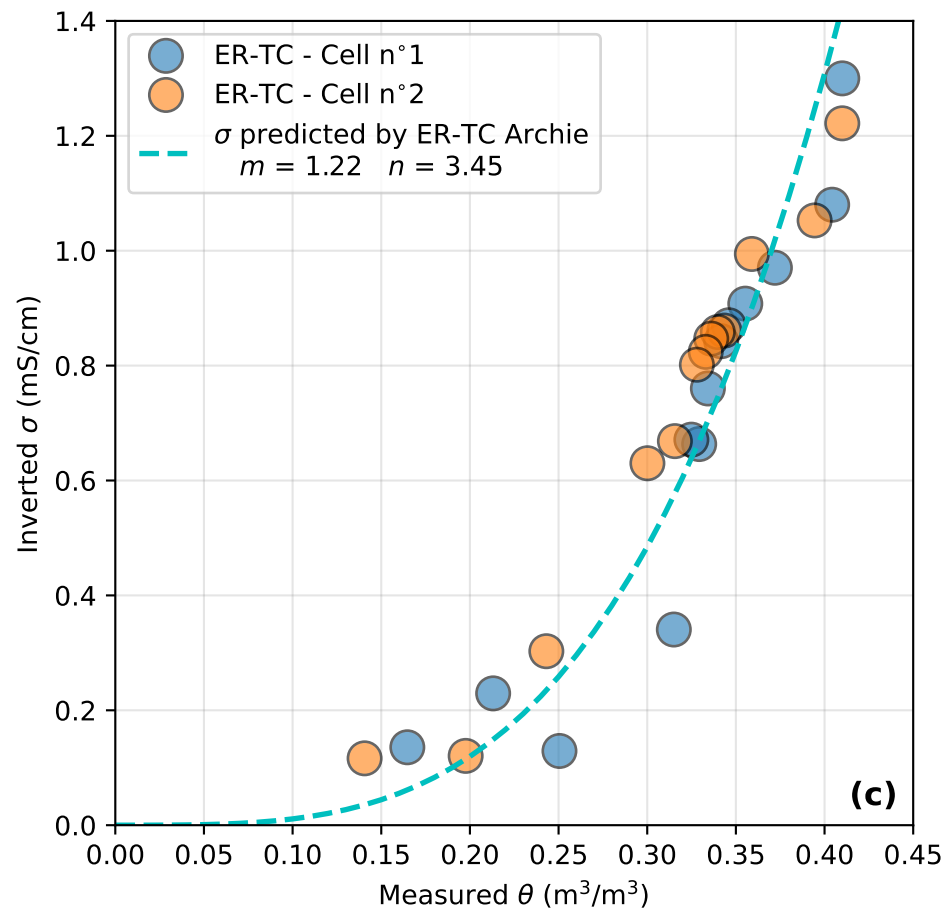

Supplement: Supplementary file 3 — Supplementary Information 3. [file 41598_2023_48100_MOESM3_ESM.zip › figs/Figure_9.pdf]

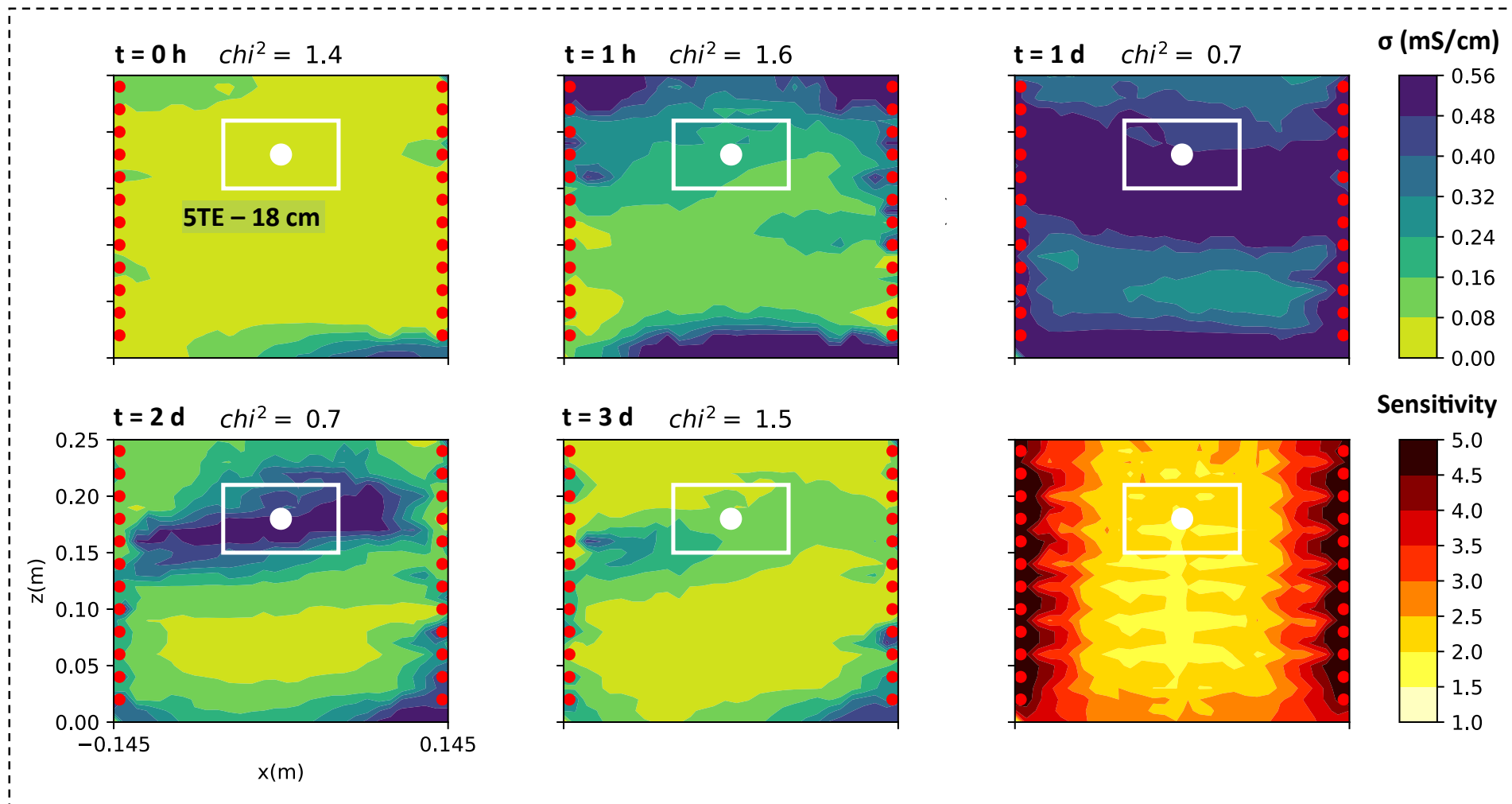

Supplement: Supplementary file 3 — Supplementary Information 3. [file 41598_2023_48100_MOESM3_ESM.zip › figs/Figure_10_part_1.pdf]

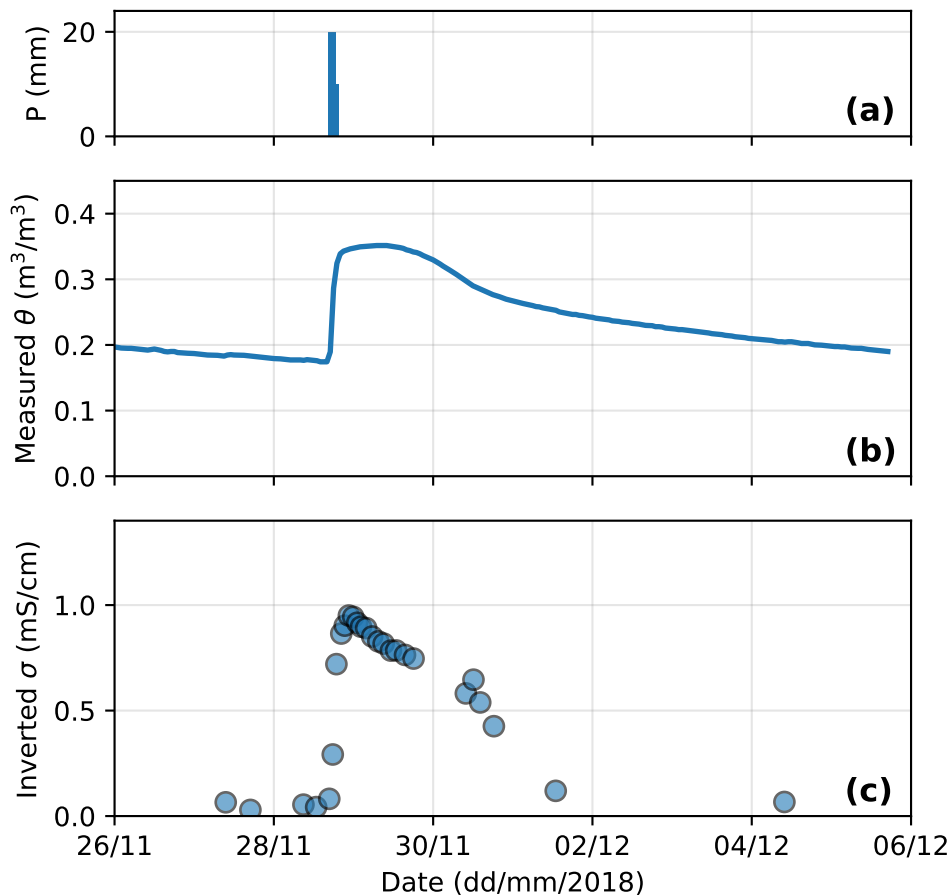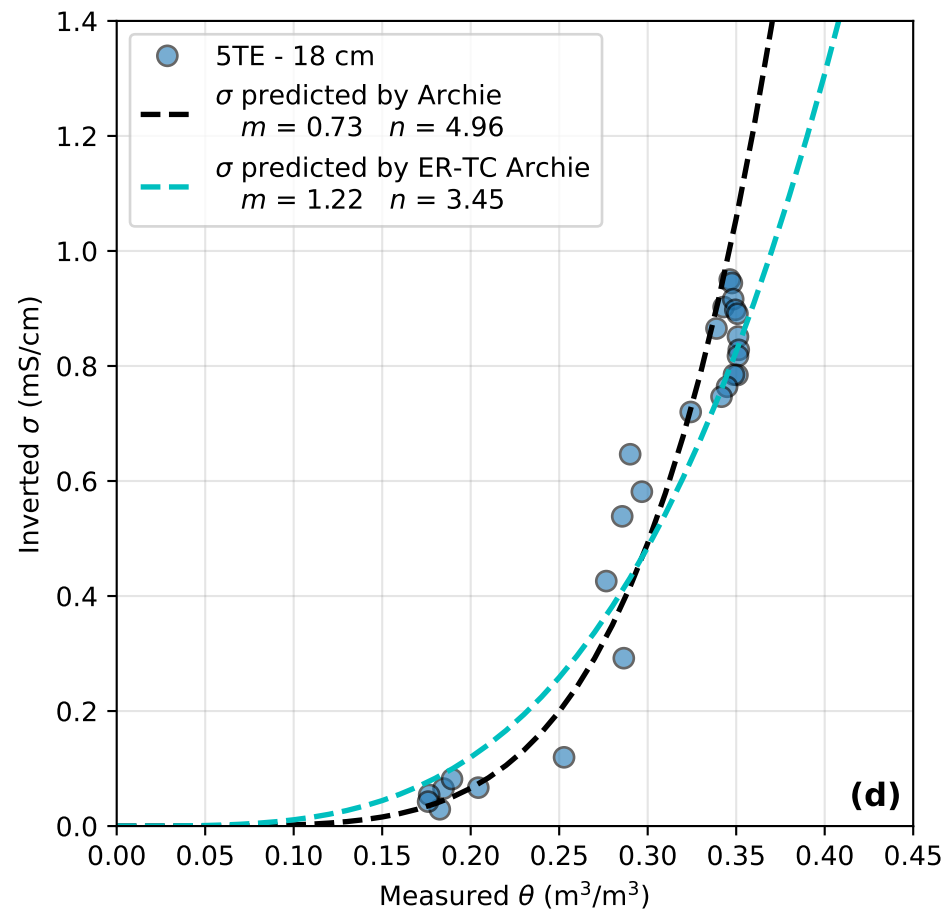

Supplement: Supplementary file 3 — Supplementary Information 3. [file 41598_2023_48100_MOESM3_ESM.zip › figs/Figure_10_part_2.pdf]

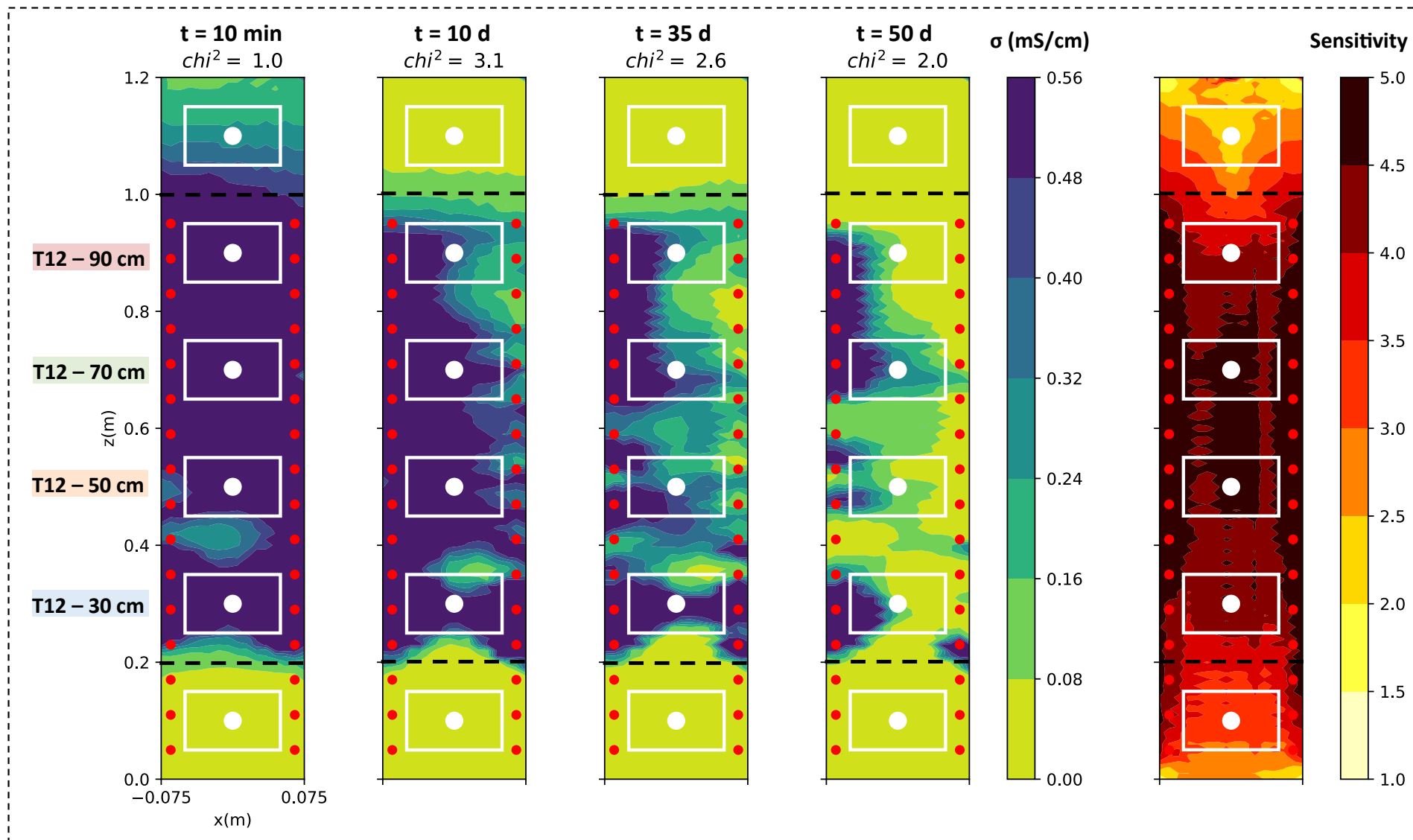

Supplement: Supplementary file 3 — Supplementary Information 3. [file 41598_2023_48100_MOESM3_ESM.zip › figs/Figure_11_part_1.pdf]

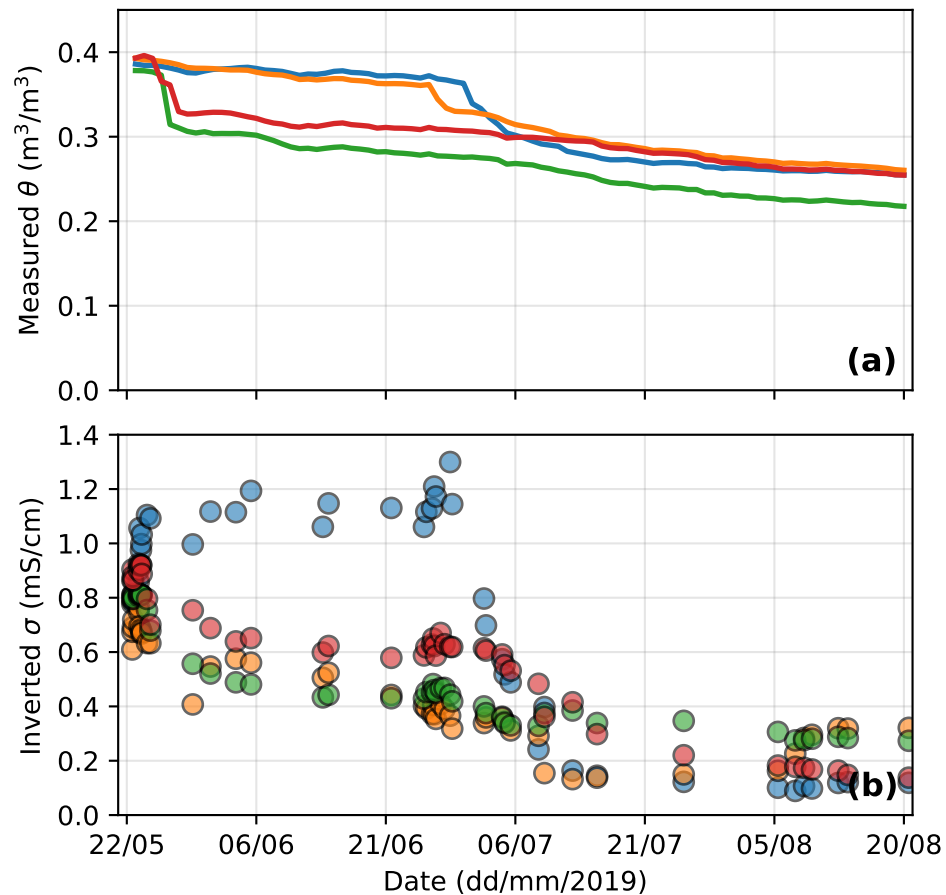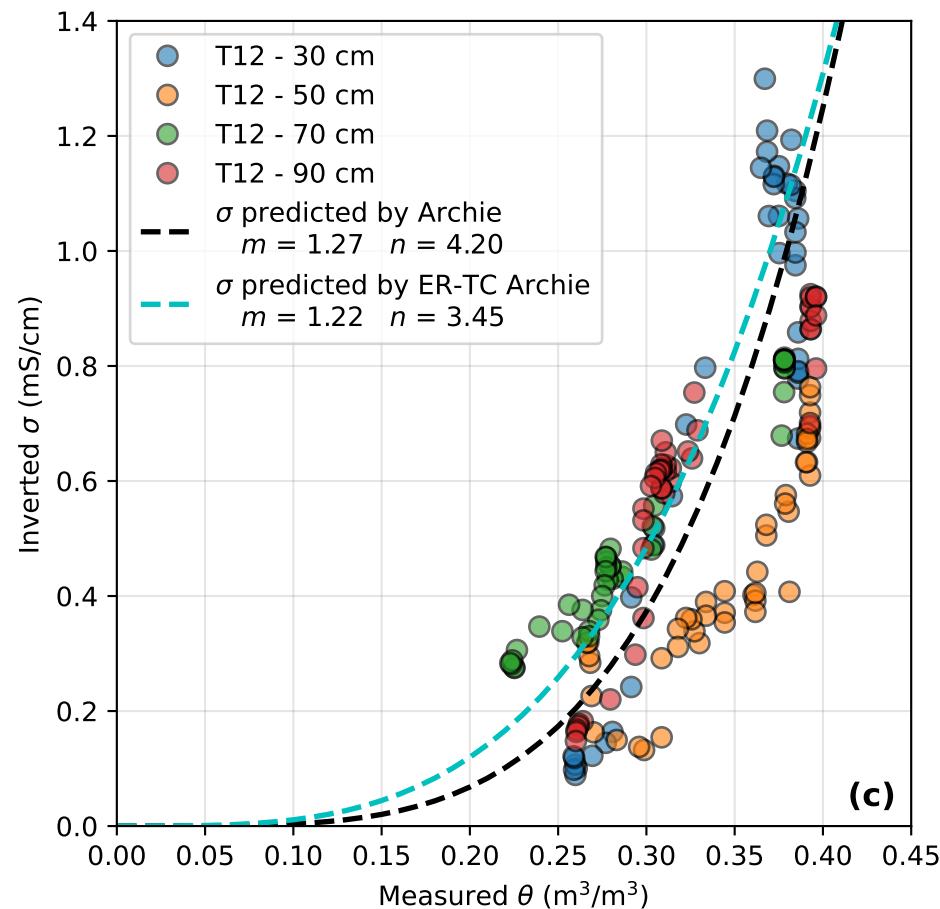

Supplement: Supplementary file 3 — Supplementary Information 3. [file 41598_2023_48100_MOESM3_ESM.zip › figs/Figure_11_part_2.pdf]

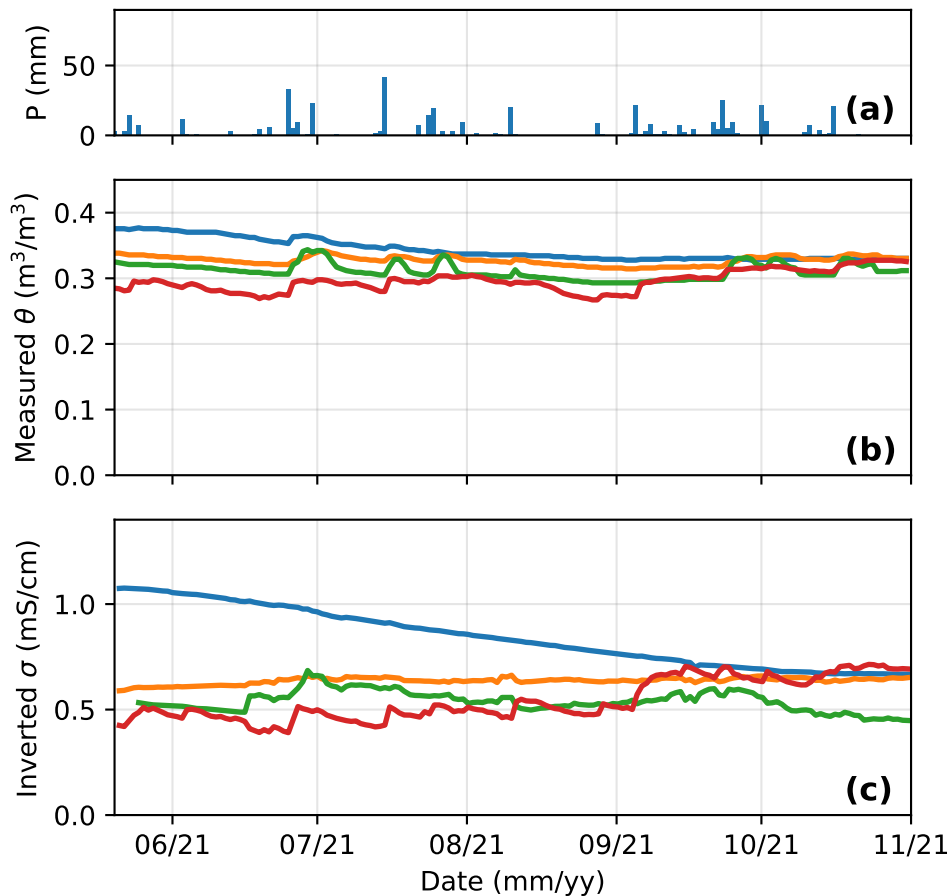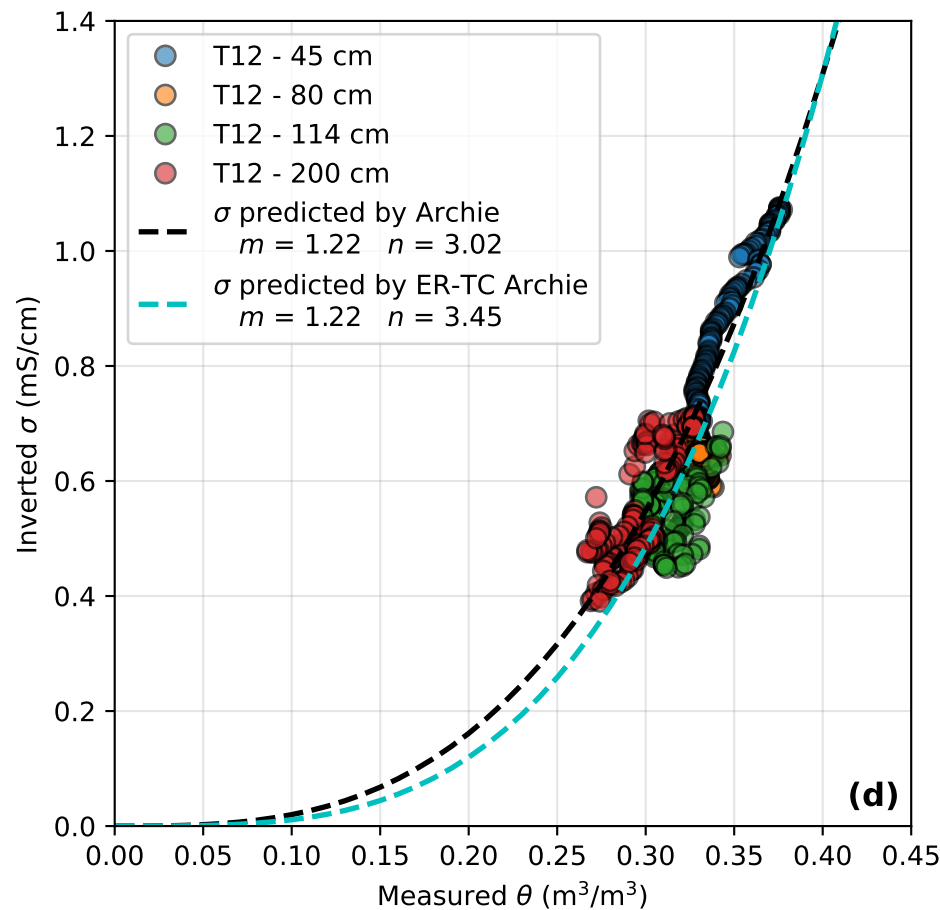

Supplement: Supplementary file 3 — Supplementary Information 3. [file 41598_2023_48100_MOESM3_ESM.zip › figs/Figure_12_part_2.pdf]

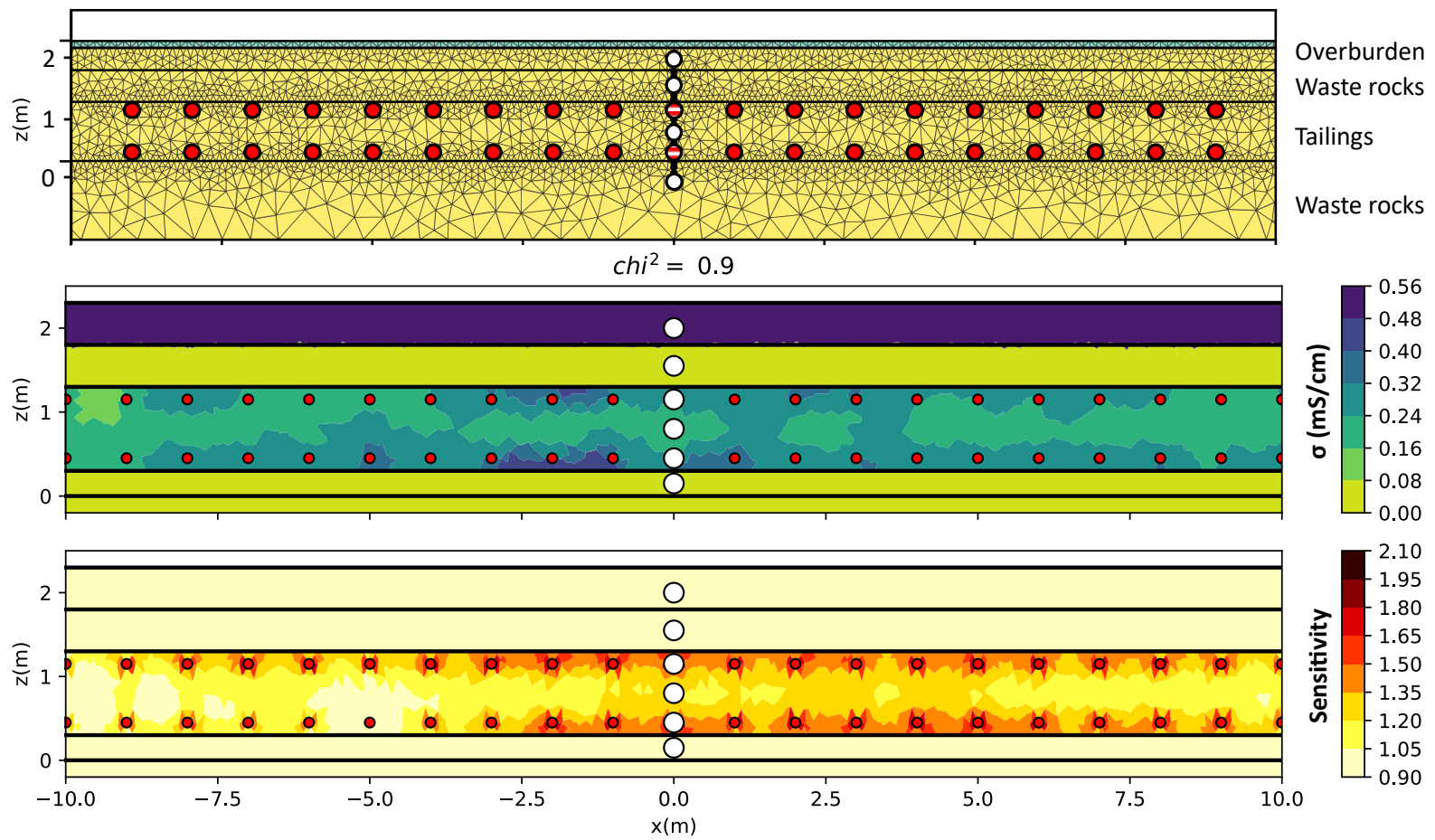

Supplement: Supplementary file 3 — Supplementary Information 3. [file 41598_2023_48100_MOESM3_ESM.zip › figs/Figure_13_part_1.pdf]

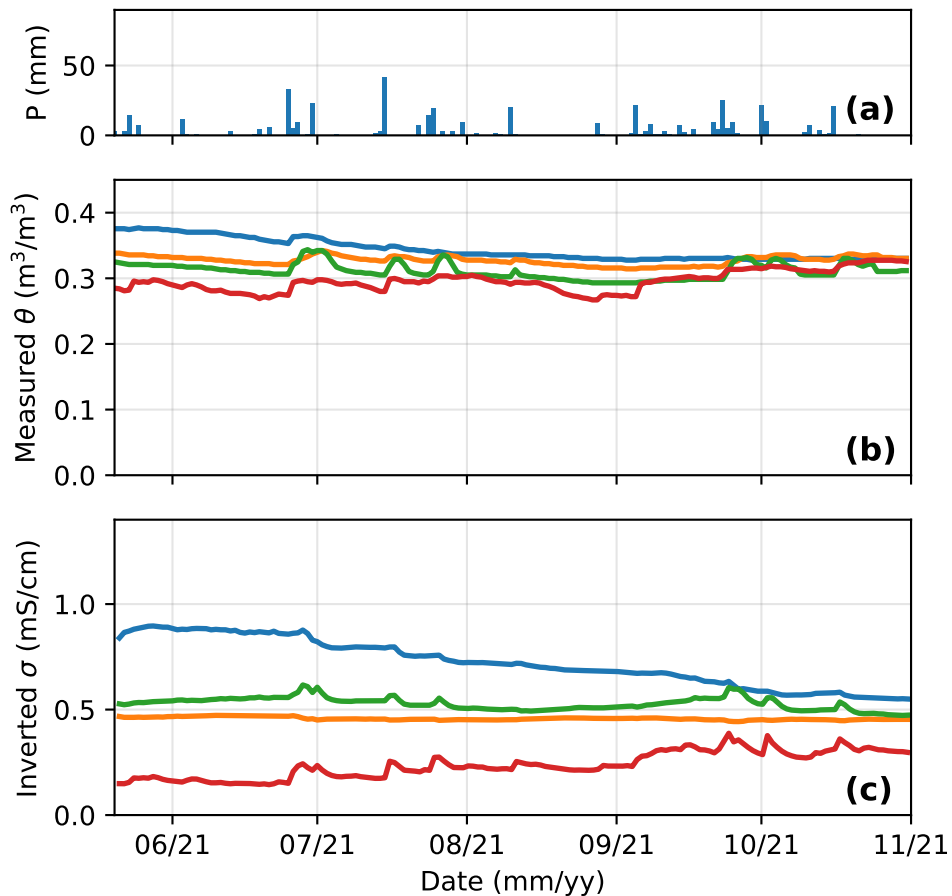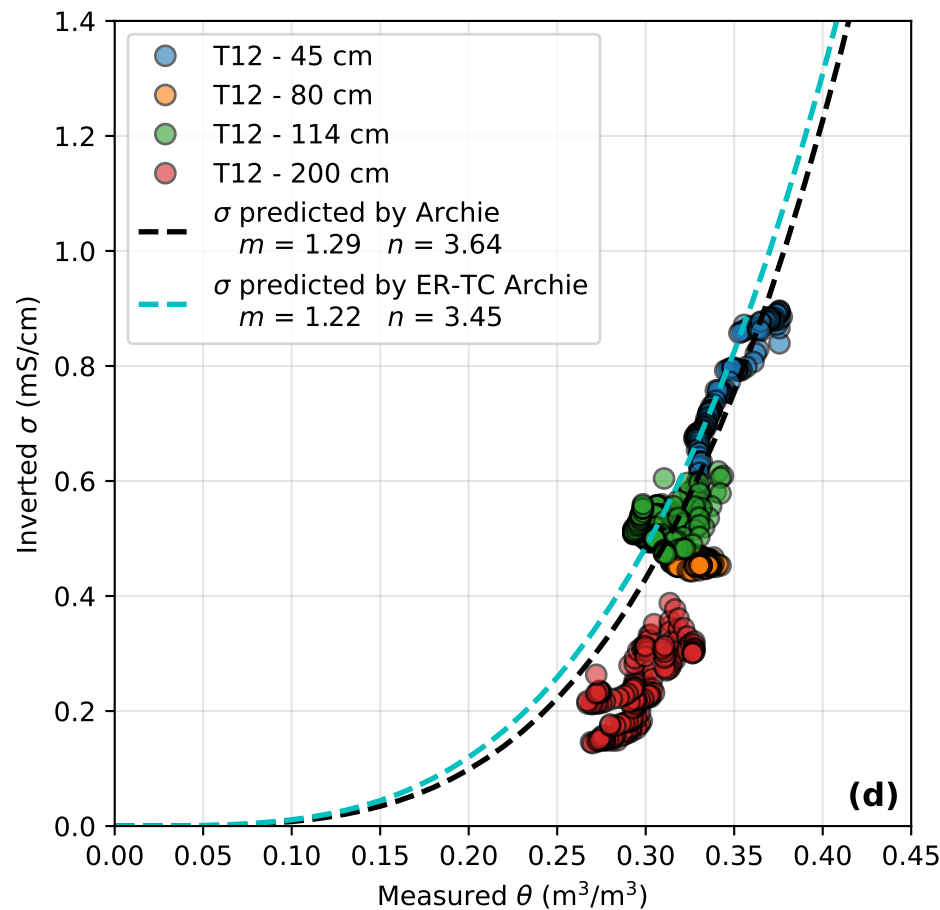

Supplement: Supplementary file 3 — Supplementary Information 3. [file 41598_2023_48100_MOESM3_ESM.zip › figs/Figure_13_part_2.pdf]

**(c)** Laboratory column

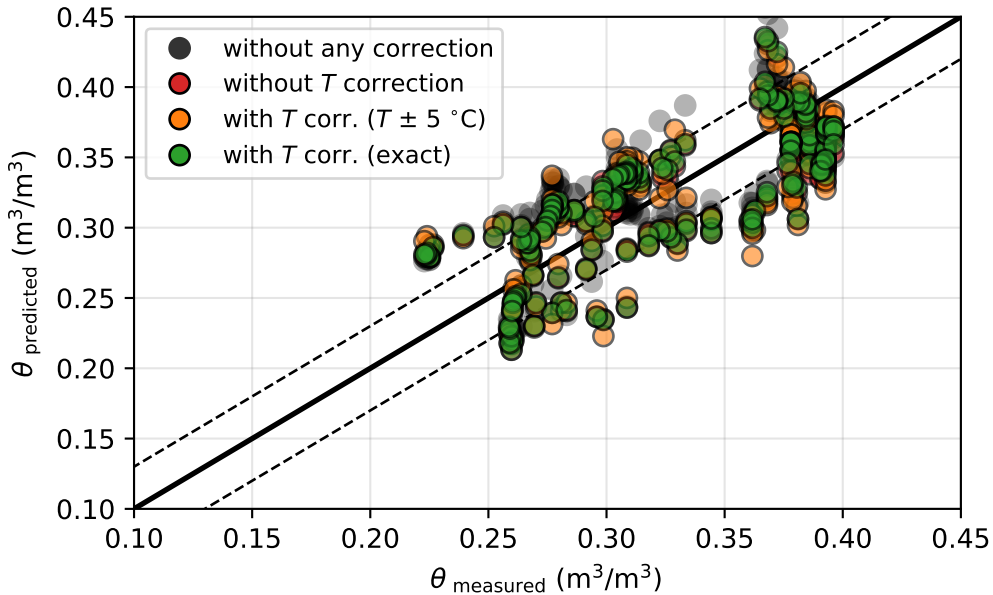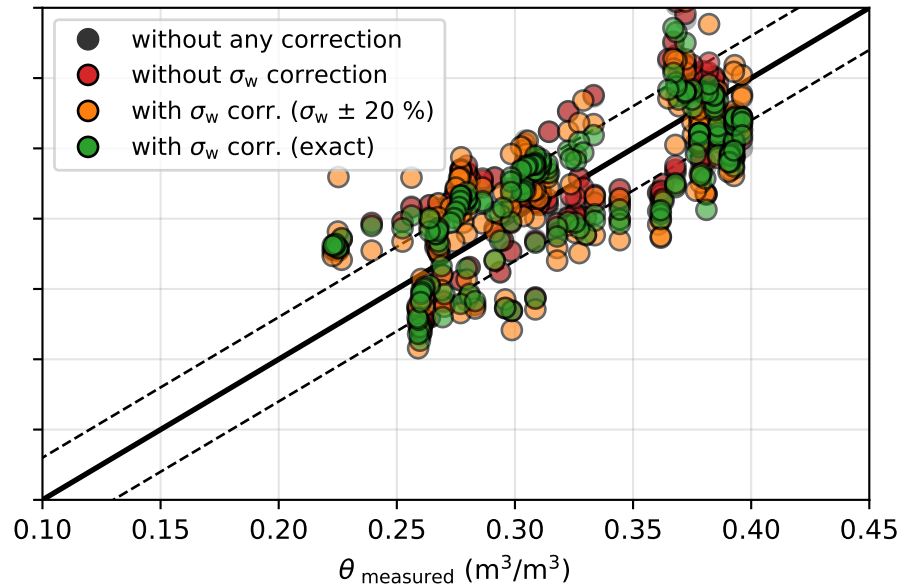

Supplement: Supplementary file 3 — Supplementary Information 3. [file 41598_2023_48100_MOESM3_ESM.zip › figs/Figure_14_part_3.pdf]

**(d)** Field experimental cover (local scale)

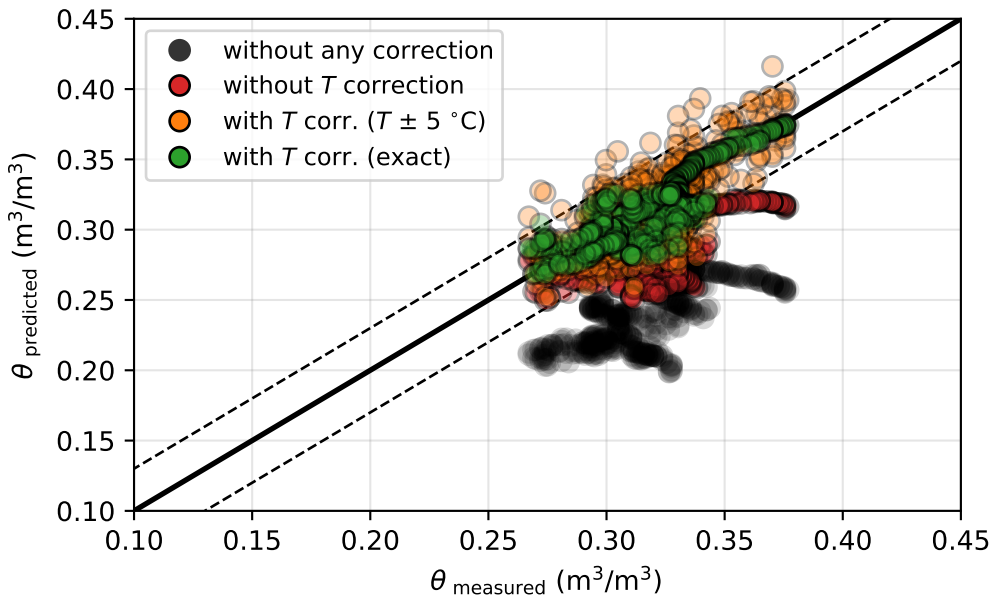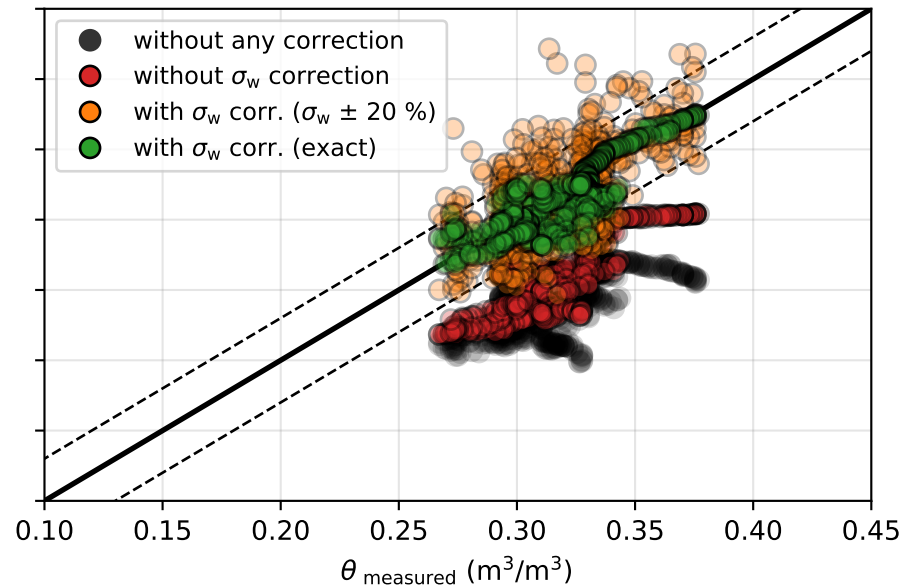

Supplement: Supplementary file 3 — Supplementary Information 3. [file 41598_2023_48100_MOESM3_ESM.zip › figs/Figure_14_part_4.pdf]

**(a)** Electrical Resistivity Tempe Cell (ER-TC)

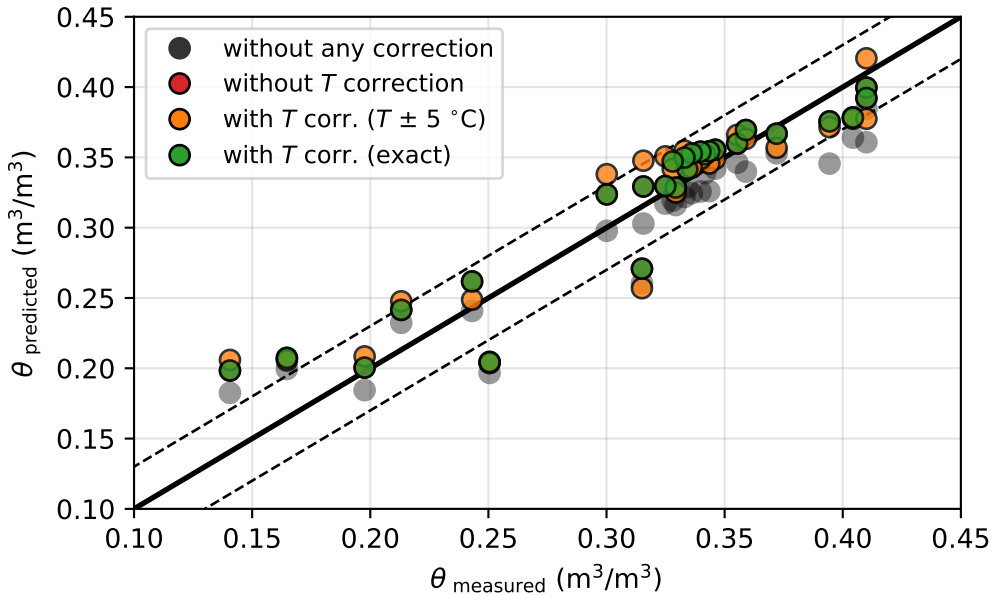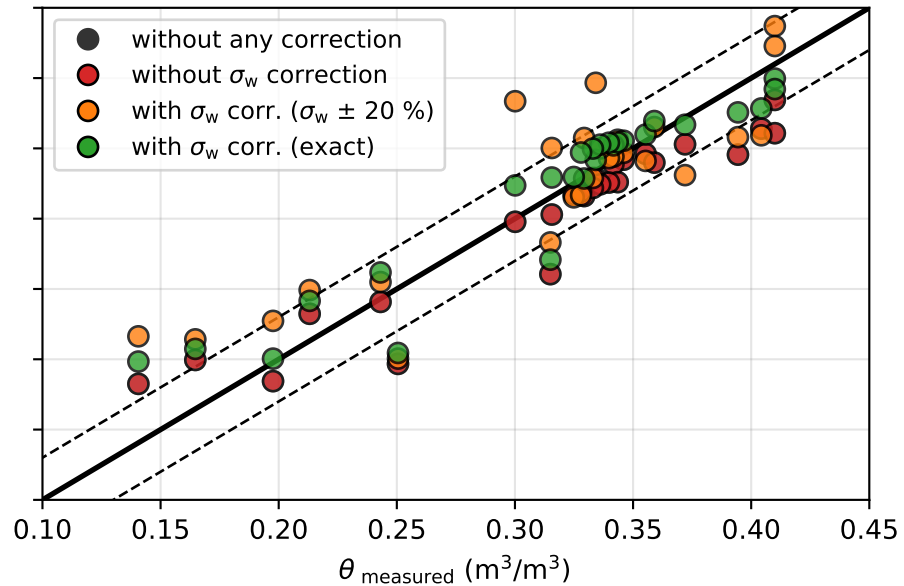

Supplement: Supplementary file 3 — Supplementary Information 3. [file 41598_2023_48100_MOESM3_ESM.zip › figs/Figure_14_part_1.pdf]

**(b)** Laboratory bucket

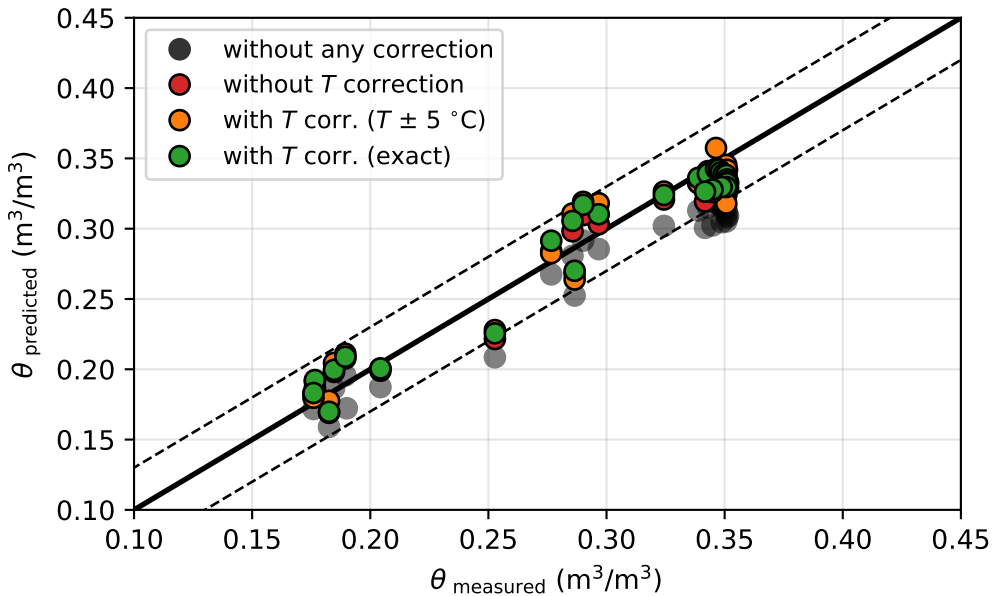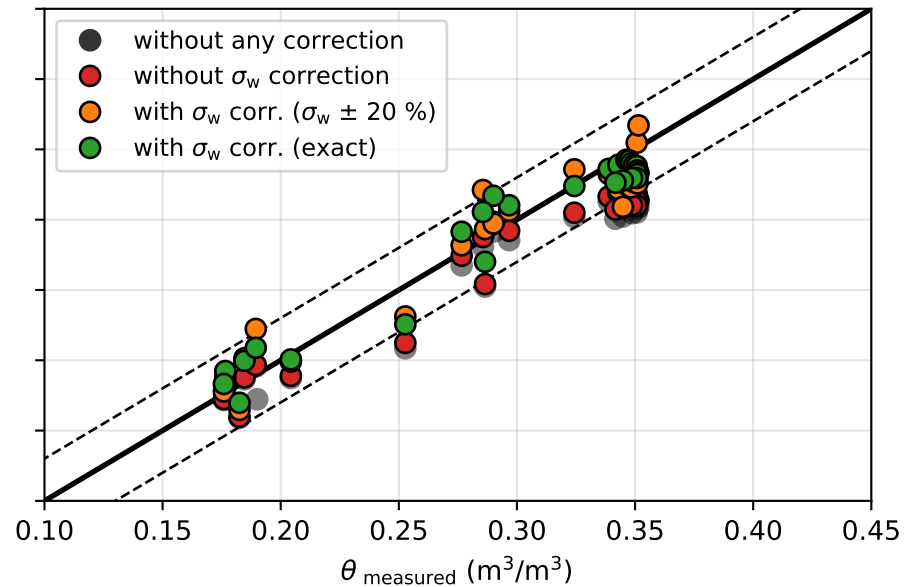

Supplement: Supplementary file 3 — Supplementary Information 3. [file 41598_2023_48100_MOESM3_ESM.zip › figs/Figure_14_part_2.pdf]

**Inversion mesh**

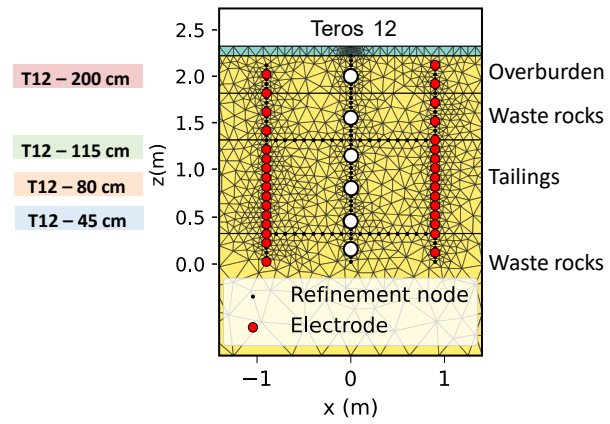

**$\sigma$  (mS/cm)**

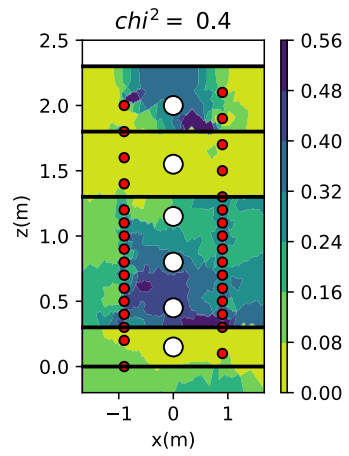

**Sensitivity**

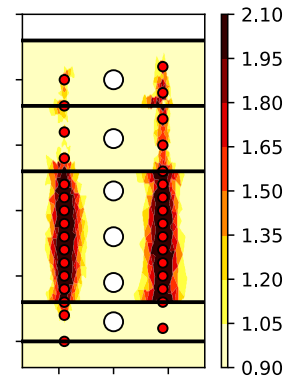

Supplement: Supplementary file 3 — Supplementary Information 3. [file 41598_2023_48100_MOESM3_ESM.zip › figs/Figure_12_part_1.pdf]

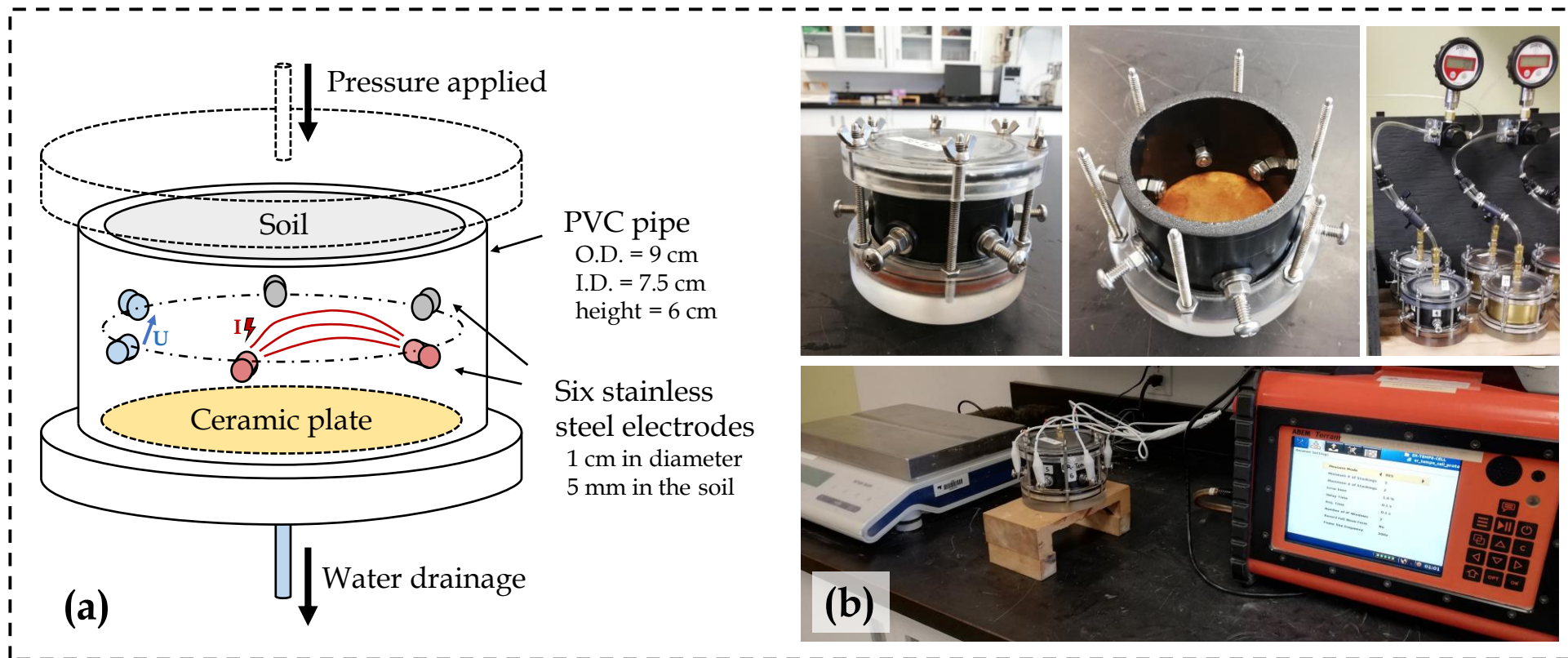

Supplement: Supplementary file 3 — Supplementary Information 3. [file 41598_2023_48100_MOESM3_ESM.zip › figs/Figure_3.pdf]

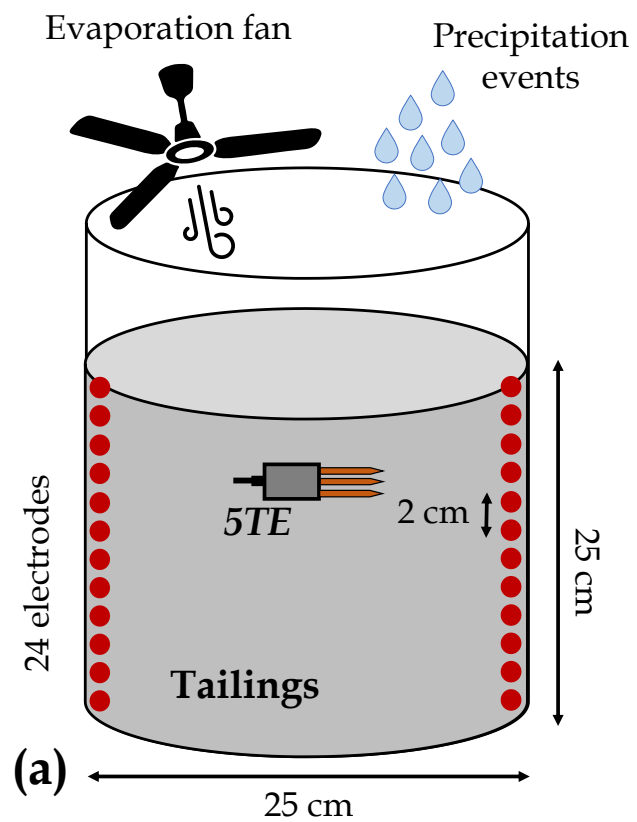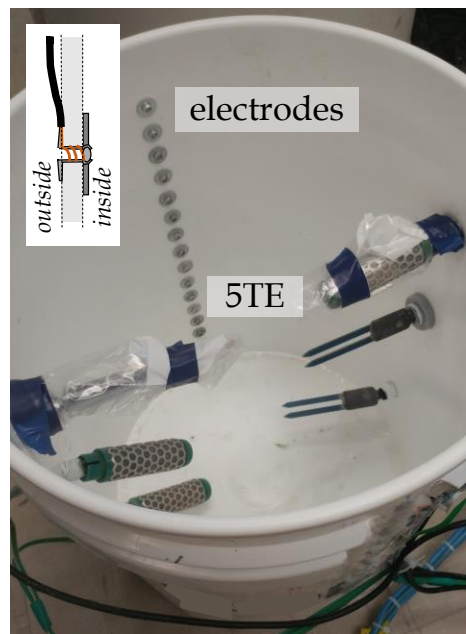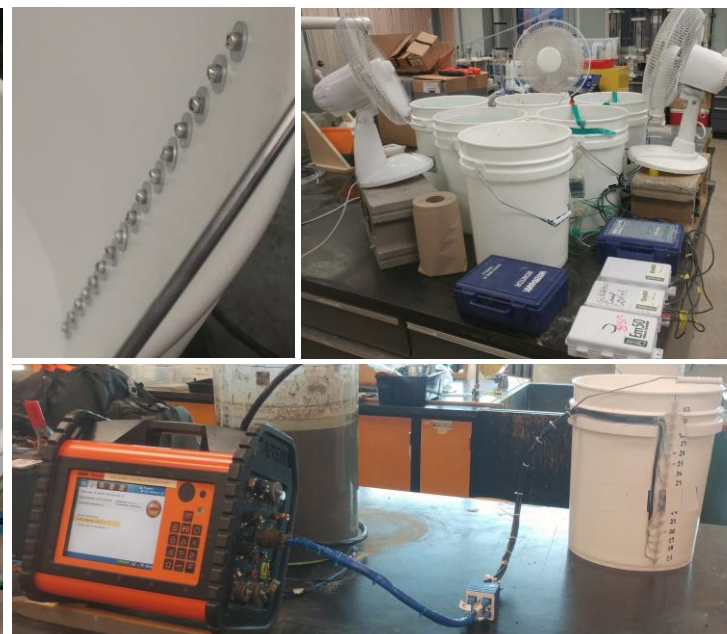

Supplement: Supplementary file 3 — Supplementary Information 3. [file 41598_2023_48100_MOESM3_ESM.zip › figs/Figure_4.pdf]

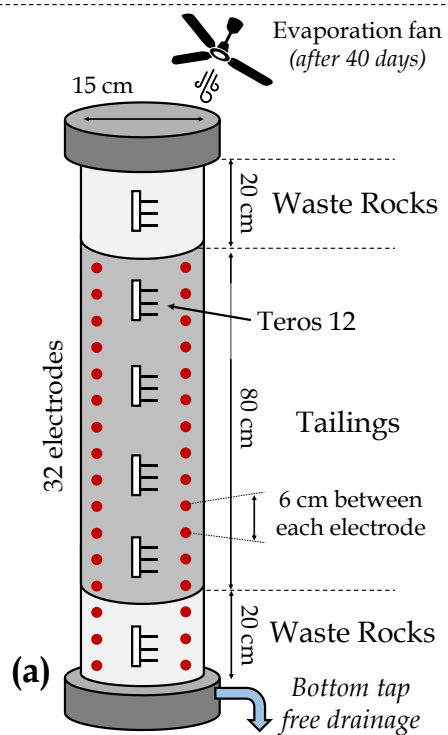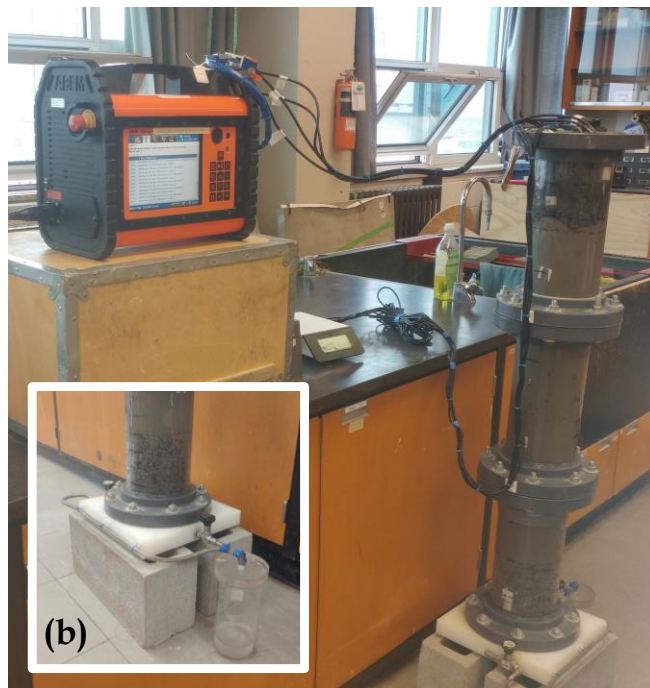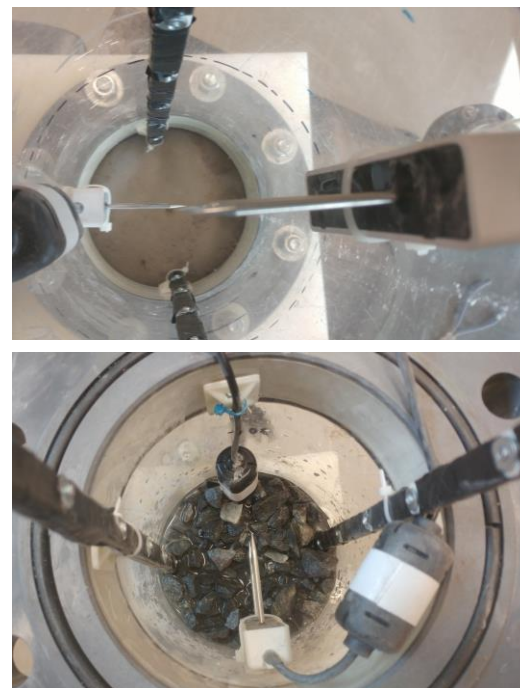

Supplement: Supplementary file 3 — Supplementary Information 3. [file 41598_2023_48100_MOESM3_ESM.zip › figs/Figure_5.pdf]

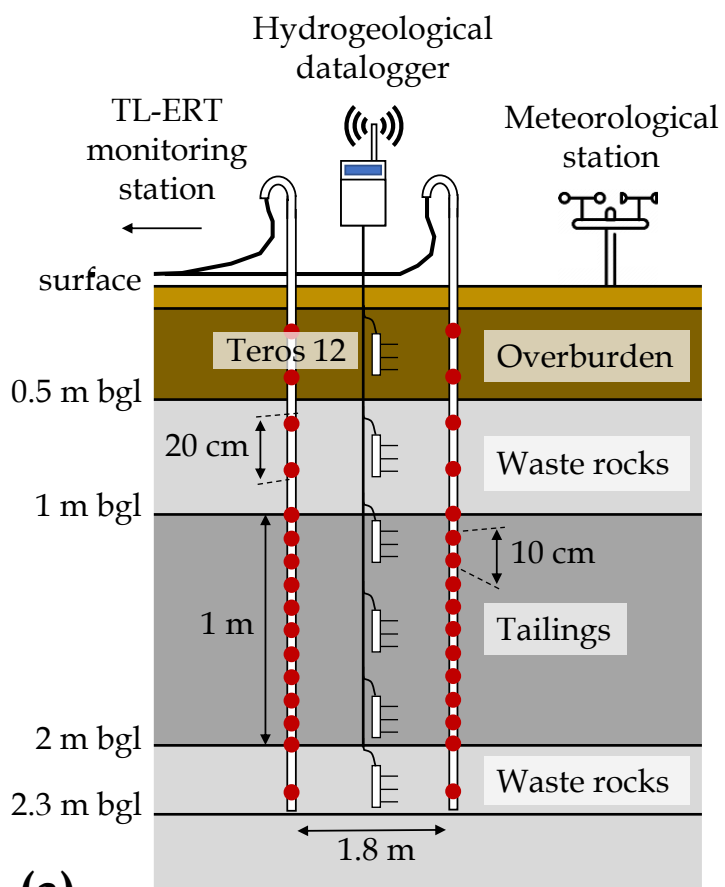

(a)

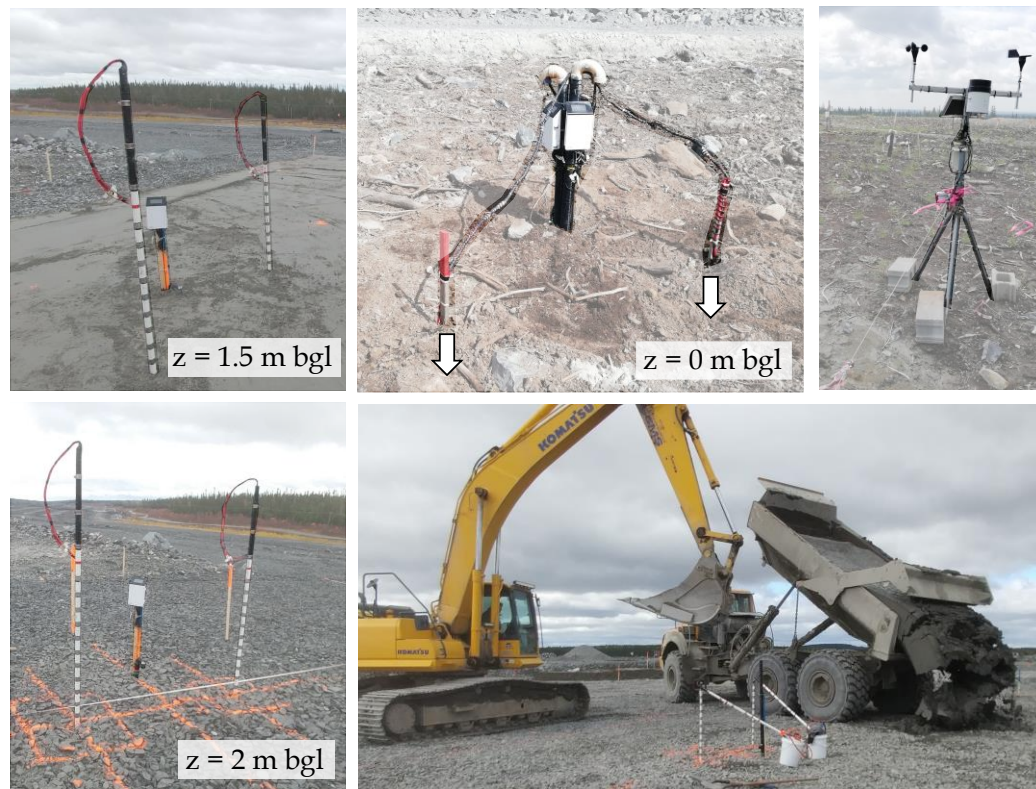

(b)

Supplement: Supplementary file 3 — Supplementary Information 3. [file 41598_2023_48100_MOESM3_ESM.zip › figs/Figure_6.pdf]

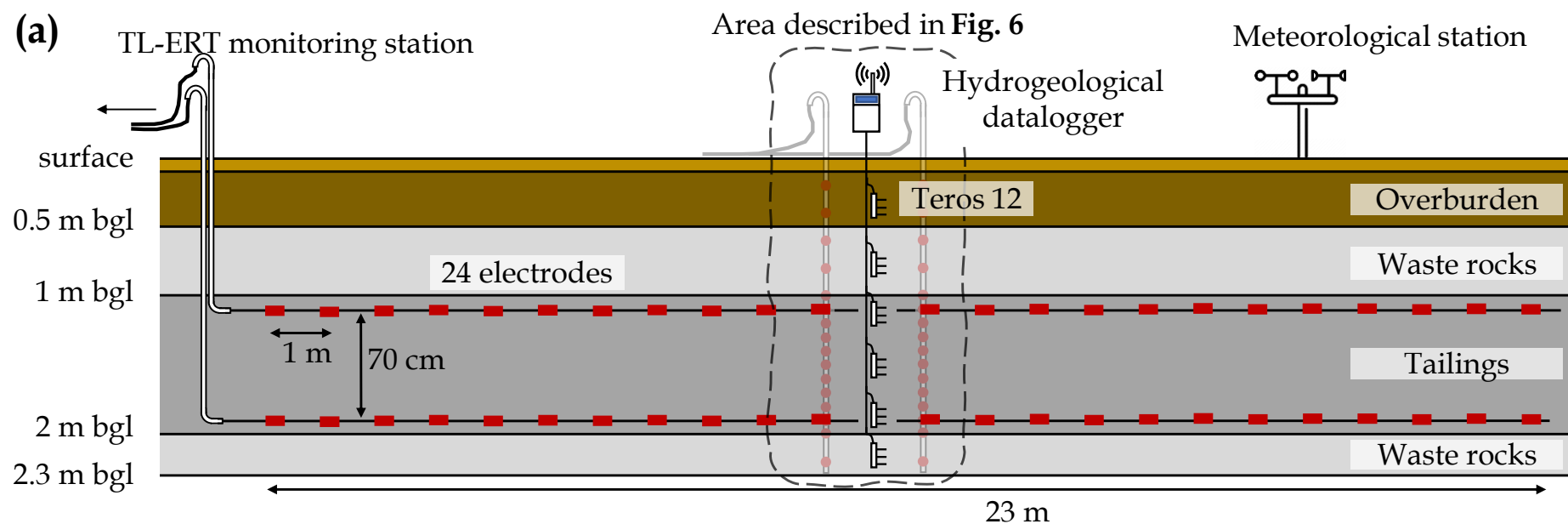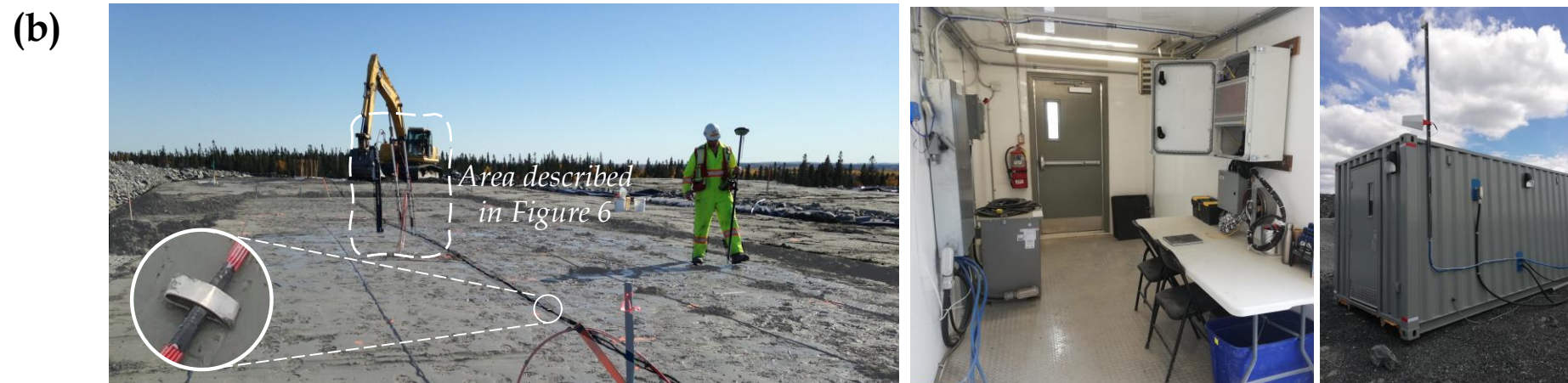

Supplement: Supplementary file 3 — Supplementary Information 3. [file 41598_2023_48100_MOESM3_ESM.zip › figs/Figure_7.pdf]
