# Supplementary material for: A multiscale accuracy assessment of moisture content predictions using time-lapse electrical resistivity tomography in mine tailings
Source: Sci Rep. 2023 Nov 27;13:20922. doi: 10.1038/s41598-023-48100-w (PMC10684595; doi:10.1038/s41598-023-48100-w)
Supplement: Supplementary file 3 — Supplementary Information 3. [file 41598_2023_48100_MOESM3_ESM.zip › figs/Figure_15.pdf]

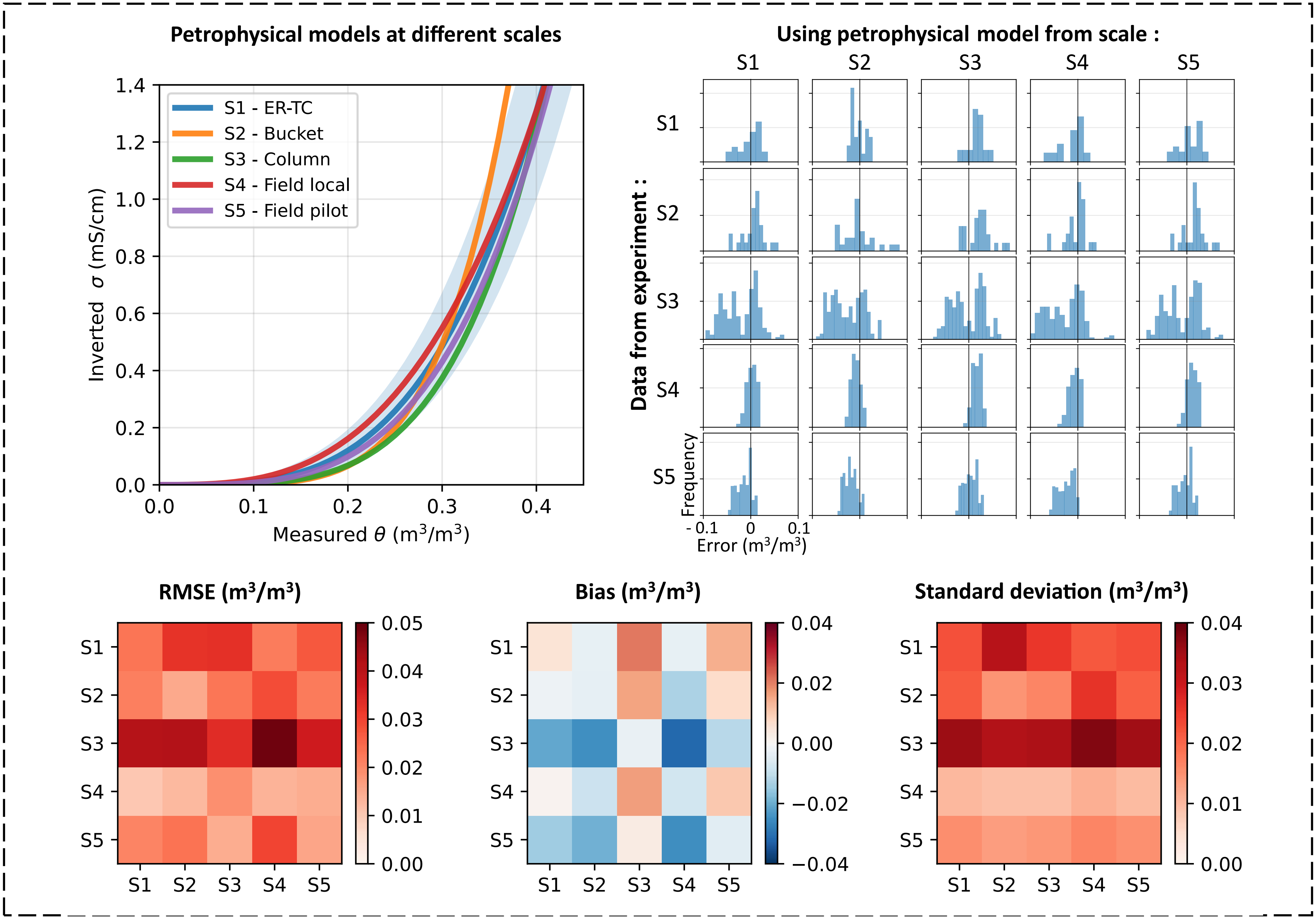

Petrophysical models at different scales
S1 - ER-TC
S2 - Bucket
S3 - Column
S4 - Field local
S5 - Field pilot
Inverted σ (mS/cm)
Measured θ (m³/m³)
Using petrophysical model from scale :
Data from experiment :
S1
S2
S3
S4
S5
Frequency
Error (m³/m³)
RMSE (m³/m³)
Bias (m³/m³)
Standard deviation (m³/m³)
